# Temporal trends and projections of gynecological cancers in China, 2007–2030

**DOI:** 10.1186/s12905-023-02384-2

**Published:** 2023-06-30

**Authors:** Rufei Duan, Hongping Zhang, Jing Yu, Sisi Deng, Haijun Yang, Yong-Tang Zheng, Yunchao Huang, Fanghui Zhao, Hongying Yang

**Affiliations:** 1 Department of Gynecology, The Third Affiliated Hospital of Kunming Medical University, Yunnan Cancer Hospital, Yunnan Cancer Center, No.519 Kunzhou road, Xishan District, Kunming, Yunnan 650118 China; 2 Key Laboratory of Animal Models and Human Disease Mechanisms, KIZ-CUHK Joint Laboratory of Bioresources and Molecular Research in Common Diseases, Kunming Institute of Zoology, Chinese Academy of Sciences, Kunming, Yunnan China; 3 School of Public Health, Kunming Medical University, Kunming, Yunnan China; 4 Department of Thoracic Surgery, The Third Affiliated Hospital of Kunming Medical University, Yunnan Cancer Hospital, Yunnan Cancer Center, Kunming, Yunnan China; 5 Department of Cancer Epidemiology, National Cancer Center / National Clinical Research Center for Cancer / Cancer Hospital, Chinese Academy of Medical Sciences and Peking Union Medical College, Beijing, China

**Keywords:** Gynecological cancer, Temporal trend, AAPC, Projection, China

## Abstract

**Background:**

Gynecological cancer will become a more important public health problem in future years but limited evidence on gynecological cancer burden in China.

**Methods:**

We extracted age-specific rate of cancer cases and deaths during 2007–2016 from the Chinese Cancer Registry Annual Report, and estimated age-specific population size using the data released by National Bureau of Statistics of China. Cancer burden were calculated by multiplying the rates with the population size. Temporal trends of the cancer cases, incidence, deaths, and mortality during 2007–2016 were calculated by JoinPoint Regression Program, and from 2017 to 2030 were projected by grey prediction model GM (1,1).

**Results:**

In China, total gynecological cancer cases increased from 177,839 to 241,800, with the average annual percentage change of 3.5% (95% CI: 2.7–4.3%) during 2007–2016. Cervical, uterine, ovarian, vulva, and other gynecological cancer cases increased by 4.1% (95% CI: 3.3–4.9%), 3.3% (95% CI: 2.6–4.1%), 2.4% (95% CI: 1.4–3.5%), 4.4% (95% CI: 2.5–6.4%), and 3.6% (95% CI: 1.4–5.9%) respectively. From 2017 to 2030, projected gynecological cancer cases are changing from 246,581 to 408,314. Cervical, vulva and vaginal cancers showed evident upward trend, while uterine and ovarian cancer cases are slightly increasing. The increases for age-standardized incidence rates were similar with that of cancer cases. Temporal trends of cancer deaths and mortality were similar with that of cancer cases and incidence during 2007–2030, except that uterine cancer deaths and mortality were declined.

**Conclusions:**

With the aging of population and other increased risk factors, the burden of gynecological cancers in China is likely to be grew rapidly in the future, comprehensive gynecological cancer control should be concerned.

**Supplementary Information:**

The online version contains supplementary material available at 10.1186/s12905-023-02384-2.

## Introduction

Gynecological cancers including the cancers of cervix, ovary, corpus uteri, vulva, vagina, and other female genital cancers. There were 570,000 cervical cancer cases and 311,000 cancer deaths were estimated annually worldwide, 382,000 corpus uteri cancer cases and 90,000 cancer deaths, and 295,000 ovarian cancer cases and 185,000 cancer deaths occurred per year globally [1]. In addition to the three main gynecological cancers, vulva and vaginal cancers combined contributed 62,000 new cancer cases and 23,000 cancer deaths in the world in 2018 [1]. Cervical cancer is well controlled in developed countries but it still the high burden in low-and-middle income countries which contributed the 80% of new cancer cases worldwide [2]. While uterine and ovarian cancers occur more frequently in developed countries [1], and were identified increasing trend in the past decades [3]. Vulva and vaginal cancer are relative rare but also showed consistently elevation globally [4].

The risk factors for ovarian and uterine cancers are breast cancer susceptibility gene (BRCA) mutations, having a family history, and other reproductive-related risk factors such as having children later or never having a full-term pregnancy, reaching menopause at an older age, and taking hormone therapy after menopauses [5]. Almost all cervical cancer is induced by human papillomavirus (HPV) infection, and about 78% of vaginal and 24% of vulva cancer are attributable to HPV [6]. Other risk factors for the vaginal-genital cancers are early age at first sexual intercourse, premature delivery, and multiple delivery. Common risk factor for all gynecological cancers is unhealthy lifestyle include smoking, alcohol use, physical inactivity, unhealthy diet, obesity, and diabetes. Cervical cancer would be well prevented by HPV vaccine and screening. The World Health Organization (WHO) has initiated the call for eliminating cervical cancer in 2018 [7]. By contrast, no feasible screening programs for both ovarian and uterine cancer at present which have no identified optimal etiological factors, current strategies to detect signs of theses cancers are limited to high-risk populations and symptomatic women [8]. Screening for the rare cancers like vulva and vaginal cancer is not cost-effectiveness at a population level, but they would been prevented at some extent by HPV vaccination during cervical cancer control.

Understanding the burden of gynecological cancer in China is expected to paving the way for establishing evidence-based policy and applicable recommendations for gynecological cancer control. Currently, limited evidence on burden and trends of gynecological cancers in China, and are only focus on the three most cancers (cervical, ovarian, and uterine) [9, 10]. We therefore conducted the study to estimate the burden, temporal trends, and projection of all gynecological cancers during 2007–2030 by cancer site, age, and geographical area.

## Materials and methods

### Data source

Gynecological cancers are represented cancers of vulva (C51), vagina (C52), cervix (C53), uterus (C54-C55), ovary (C56), and other gynecological cancer (C57-C58) based on the International Classification of Diseases, 10th revision (ICD-10). Age-specific rate of cancer cases and deaths during 2007–2016 were obtained from the published Chinese Cancer Registry Annual Report (2010–2019) published by the National Central Cancer Registry of China (NCCR). The NCCR of China was founded in 2002, acting as the national bureau for the management of cancer registration [11]. The cancer data in 2016 were collected from 487 cancer registries, covered 382 million persons (194 million males and 188 million females), accounting for about 27.60% of the national population [12]. Age-specific population size from 2007 to 2016 was estimated using the data released by National Bureau of Statistics of China.

### Statistical analysis

Gynecological cancer cases and deaths were calculated by multiplying age-specific rates with the estimated population size. The Segi’s population was applied to calculate age-standardized rate. The corresponding estimates of 95% confidence interval (CI) of the cancers were calculated. Temporal trends of the cancer cases, incidence, deaths, and mortality from 2007 to 2016 were calculated by JoinPoint Regression Program. The analysis identifies periods with distinct linear slopes that can be connected by join-points denoting trend changes and determine the number of join-points that should be used to best describe trends in the data [13]. Average annual percentage change (AAPC) and the corresponding 95% CIs were calculated. The cancer cases, incidence, deaths, and mortality were considered increasing if the 95% CI of AAPC was entirely above zero while decreasing if the 95% CI of AAPC was below zero, otherwise, they were considered stable. 6) Cancer cases, incidence, deaths, and mortality were further projected from 2017 to 2030 by grey prediction model GM (1,1). Grey GM (1,1) model is one of the homogeneous exponential growth models based on the accumulation generation sequence and the least-squares method [14]. It applies the accumulative generation operation (AGO) to the primary data and proceeds to solve the resulting differential equation. Then it performs an inverse AGO and calculates the predicted values of the primary data. Grey prediction model GM (1,1) showed a high fitting accuracy (86.25%) on cancer prediction when compared with other predicting methods, and it has been used for hepatocellular carcinoma, cervical cancer, and other cancers temporal trend projections [15, 16]. Data management and analysis were conducted on Microsoft Excel 2010, R 3.6.2, and JoinPoint Regression Program 4.7.0.

## Results

### Burden of gynecological cancers in China

Table 1, Additional files S1 to S2 presented the gynecological cancer cases and incidence in 2016. 241,800 gynecological cancer cases were estimated, including 113,256 cervical, 66,228 uterine, 53,490 ovarian, 4,437 other gynecological, 2,827 vulva, and 1,561 vaginal cancers. The age-standardized incidence rate (ASIR) of all gynecological cancers is 24.20 per 100,000 persons, the ASIR for cervical, uterine, ovarian, other gynecological, vulva, and vaginal cancers were 11.39, 6.52, 5.41, 0.46, 0.27, and 0.15 per 100,000 persons respectively. Urban and rural areas contributed 140,409 and 101,391 cancer cases respectively, ASIR of ovarian cancer is slightly higher in urban compared with rural areas (5.94 versus 4.89 per 100,000 persons). 61.5% of gynecological cancers were diagnosed in women aged 45–64 years, pecked at 50–54 years, the incidence is continuous increasing and peak at 50–54 years.

**Table 1:** Gynecological cancer cases, incidence, deaths and mortality in China, 2016

Cancer site (ICD-10)	Cases	ASIR (per 100,000 persons)		Deaths	ASMR (per 100,000 persons)
Total	241,800	24.20		79,170	7.53
**Vulva (C51)**	2827	0.27		999	0.09
**Vagina (C52)**	1561	0.15		616	0.06
**Cervix (C53)**	113,256	11.39		35,353	3.40
**Uterus (C54-C55)**	66,228	6.52		15,891	1.52
**Ovary (C56)**	53,490	5.41		24,953	3.40
**Other gynecological cancers (C57-C58)**	4437	0.46		1357	0.13
Urban areas	140,409	24.48		45,222	7.73
**Vulva (C51)**	1687	0.29		611	0.10
**Vagina (C52)**	953	0.17		409	0.07
**Cervix (C53)**	63,145	10.89		19,037	3.25
**Uterus (C54-C55)**	38,577	6.69		8357	1.43
**Ovary (C56)**	33,209	5.94		15,909	2.73
**Other gynecological cancers (C57-C58)**	2837	0.50		897	0.16
Rural areas	101,391	23.91		33,948	7.33
**Vulva (C51)**	1140	0.25		388	0.08
**Vagina (C52)**	609	0.14		207	0.04
**Cervix (C53)**	50,111	11.89		16,316	3.54
**Uterus (C54-C55)**	27,651	6.36		7534	1.61
**Ovary (C56)**	20,281	4.87		9044	1.97
**Other gynecological cancers (C57-C58)**	1600	0.41		460	0.10

Abbreviations: ICD-10, International Classification of Diseases 10th revision; ASIR, age-standardized incidence rate;

ASMR, age-standardized mortality rate

Table 1, Additional File Figures S3 to S4 presented the gynecological cancer deaths and mortality in 2016. 79,170 gynecological cancer deaths were estimated, with the age-standardized mortality rate (ASMR) 7.53 per 100,000 persons. Cervical cancer is the highest burden (35,353 cancer deaths, ASMR: 3.40 per 100,000 persons), followed by cancers of ovary (24,953, 2.35), uterine (15,891, 1.52), other gynecological (1,357, 0.13), vulva (999, 0.09), and vaginal (616, 0.06). Urban and rural areas contributed 45,222 and 33,948 gynecological cancer deaths respectively, the ASMR of ovarian cancer being slightly higher in urban than in rural areas (2.73 versus 1.97 per 100,000 persons). 64.7% of gynecological cancer deaths occurred among women aged 45–69 years, with the peak among those aged 50–54 years, while the ASMRs for total cancers showed a continuous increasing trend by age.

### Trends of gynecological cancers in China

Table 2, Additional File Figures S5 to S6, Supplementary Tables S1 to S6 presented the trends of gynecological cancer cases and incidence during 2007–2016. Total cancer cases increased from 177,839 to 241,800, with the AAPC of 3.5% (95% CI: 2.7–4.3%). The ASIR elevated from 19.97 to 24.20 per 100,000 persons (AAPC: 2.4%, 95% CI: 1.9–2.9%). Cervical cancer cases increased by 4.1% (95% CI: 3.3–4.9%), and incidence rose by 3.4% (95% CI: 3.1–3.7%) annually, the increases were most evident among women over 45 years. Uterine cancer cases and incidence elevated by 3.3% (95% CI: 2.6–4.1%) and 2.0% (1.1–2.9%) per year respectively, and the highest increases were identified at the age of 45–49 and 60–69. A raising trend of ovarian cancer burden was identified with the AAPC of 2.4% (95% CI: 1.4–3.5%) for cases and 0.9% (95% CI: 0.2–1.7%) for incidence, particularly among those aged 25–29 and 60–69 years. Vulva cancer have risen by 4.4% (95% CI: 2.5–6.4%) for cases and 2.5% (0.6–4.5%) for incidence, with the most rapid increase happening among those over 60 years. Vaginal cancer showed a growing trend but insignificant. Other gynecological cancer cases rose by 3.6% (95% CI: 1.4–5.9%) but the increase of incidence was insignificant. Cervical and uterine cancers growing faster in urban, while vulva and ovarian cancers increased more evident in rural areas.

**Table 2:** Trends analysis of gynecological cancer cases and incidence in China, 2007–2016

Cancer site (ICD-10)	Cancer cases				ASIR (per 100,000 persons)		
	2007	2016	AAPC (95% CI)		2007	2016	AAPC (95% CI)
Total	177,839	241,800	3.5*(2.7~4.3)		19.97	24.20	2.4*(1.9~2.9)
**Vulva (C51)**	1833	2827	4.4*(2.5~6.4)		0.21	0.27	2.5*(0.6~4.5)
**Vagina (C52)**	1378	1561	3.2(-0.3~6.8)		0.15	0.15	1.6(-1.3~4.6)
**Cervix (C53)**	78,510	113,256	4.1*(3.3~4.9)		8.50	11.39	3.4*(3.1~3.7)
**Uterus (C54-C55)**	50,510	66,228	3.3*(2.6~4.1)		5.68	6.52	2.0*(1.1~2.9)
**Ovary (C56)**	43,023	53,490	2.4*(1.4~3.5)		5.11	5.41	0.9*(0.2~1.7)
**Other gynecological cancers (C57-C58)**	2585	4437	3.6*(1.4~5.9)		0.32	0.46	1.8(-0.6~4.3)
Urban areas	92,139	140,409	4.3*(3.2~5.4)		22.30	24.48	1.0*(0.6~1.4)
**Vulva (C51)**	1252	1687	2.8*(0.3~5.3)		0.31	0.29	-0.8(-3.4~1.9)
**Vagina (C52)**	763	953	3.6*(0.8~6.4)		0.18	0.17	0.5(-1.9~3)
**Cervix (C53)**	34,510	63,145	6.2*(5.1~7.3)		7.97	10.89	3.5*(1.8~5.4)
**Uterus (C54-C55)**	26,234	38,577	3.9*(2.5~5.4)		6.45	6.69	0.5(-0.1~1.1)
**Ovary (C56)**	27,560	33,209	1.6(-0.7~4)		6.93	5.94	-1.6*(-2.6~-0.7)
**Other gynecological cancers (C57-C58)**	1820	2837	3.6*(1.3~6.1)		0.47	0.50	-0.2(-1.8~1.5)
Rural areas	85,699	101,391	3.8*(0.5~7.3)		17.65	23.91	4.0*(3.1~4.9)
**Vulva (C51)**	581	1140	7.0*(4~10.1)		0.11	0.25	8.1*(5.4~10.8)
**Vagina (C52)**	615	609	2.6(-2.8~8.2)		0.12	0.14	2.9(-2~8.1)
**Cervix (C53)**	43,999	50,111	1.5*(0.3~2.6)		9.03	11.89	3.2*(2.4~4.1)
**Uterus (C54-C55)**	24,276	27,651	2.6*(0.9~4.3)		4.91	6.36	3.9*(1.8~6)
**Ovary (C56)**	15,463	20,281	3.4*(1.6~5.2)		3.30	4.87	4.7*(2.4~7.1)
**Other gynecological cancers (C57-C58)**	765	1600	3.1(-2~8.5)		0.17	0.41	4.1(-1.4~9.9)

Abbreviations: ICD-10, International Classification of Diseases 10th revision; ASIR, age-standardized incidence rate; AAPC: average annual percentage change; CI: confidence interval

*: statistically significance

Table 3, Additional File Figures S7 to S8, Supplementary Tables S1 to S6 showed the trends of gynecological cancer deaths and mortality during 2007–2016. Total cancer deaths increased from 59,169 to 79,170 (AAPC: 3.1%, 95% CI:1.4–4.8%), and mortality elevated from 6.45 to 7.53 per 100,000 persons (AAPC: 2.0%, 95% CI:1.4–2.7%). Cervical cancer deaths and mortality increased by 5.1% (95% CI: 3.1-7.0%) and 4.1% (95% CI:1.4–6.8%) per year respectively, the evident increases among women aged 45–54, and over 60 years. Uterine cancer deaths and mortality were declined by 1.7% and 2.8% per year respectively. Ovarian cancer elevated by 4.5% (95% CI: 3.2–5.8%) for deaths and 2.4% (1.9–2.9%) for mortality annually respectively, the increases mainly happened in those aged 45–49 and over 60. Growing trends on vulva cancer death and mortality were identified but not statistically significant. Vaginal cancer deaths rose by 7.0% per year while the increasing of mortality is insignificant. Other gynecological cancer deaths elevated by 9.7% and 7.3% per year and most evident at 65–69 years. Urban and rural were identified with similar trends of gynecological cancer deaths and mortality, except vaginal and cervical cancer deaths that increased faster in urban areas.

**Table 3:** Trends analysis of gynecological cancer deaths and mortality in China, 2007–2016

Cancer site (ICD-10)	Cancer deaths				ASMR (per 100,000 persons)		
	2007	2016	AAPC (95% CI)		2007	2016	AAPC (95% CI)
Total	59,169	79,170	3.1*(1.4~4.8)		6.45	7.53	2.0*(1.4~2.7)
**Vulva (C51)**	930	999	4.3(-0.8~9.6)		0.11	0.09	1.3(-3.7~6.6)
**Vagina (C52)**	370	616	7.0*(2.8~11.3)		0.05	0.06	3.5(-0.6~7.8)
**Cervix (C53)**	22,457	35,353	5.1*(3.1~7)		2.37	3.40	4.1*(1.4~6.8)
**Uterus (C54-C55)**	18,639	15,891	-1.7*(-3.1~-0.3)		2.00	1.52	-2.8*(-4.5~-1.1)
**Ovary (C56)**	16,355	24,953	4.5*(3.2~5.8)		1.87	3.40	2.4*(1.9~2.9)
**Other gynecological cancers (C57-C58)**	418	1357	9.7*(4.8~14.8)		0.05	0.13	7.3*(2.3~12.5)
Urban areas	24,390	45,222	6.4*(3.9~9.1)		5.90	7.73	2.6*(1.9~3.4)
**Vulva (C51)**	552	611	1.9(-1~4.8)		0.14	0.10	-1.2(-5.5~3.4)
**Vagina (C52)**	148	409	9.0*(3.5~14.9)		0.05	0.07	3.5(-0.5~7.6)
**Cervix (C53)**	6524	19,037	11.8*(10.1~13.6)		1.52	3.25	8.1*(6.6~9.7)
**Uterus (C54-C55)**	6779	8357	1.4*(0.4~2.5)		1.66	1.43	-1.8*(-3.5~-0.1)
**Ovary (C56)**	10,059	15,909	4.3*(1~7.8)		2.46	2.73	0.8(-0.3~1.8)
**Other gynecological cancers (C57-C58)**	327	897	6.5*(1~12.3)		0.08	0.16	2.4(-3.5~8.7)
Rural areas	34,779	33,948	0.7(-0.9~2.3)		6.99	7.33	1.4(-0.1~2.9)
**Vulva (C51)**	378	388	5.2(-2.8~13.8)		0.08	0.08	4.8(-2.9~13.1)
**Vagina (C52)**	222	207	3.1(-4.8~11.7)		0.05	0.04	2.5(-5.3~11)
**Cervix (C53)**	15,932	16,316	0.1(-2.3~2.5)		3.22	3.54	1(-1.5~3.5)
**Uterus (C54-C55)**	11,860	7534	-4.2*(-6.4~-2)		2.35	1.61	-3.6*(-5.9~-1.3)
**Ovary (C56)**	6296	9044	4.6*(2.9~6.4)		1.28	1.97	5.1*(3.2~7)
**Other gynecological cancers (C57-C58)**	91	460	14.2*(3.8~25.7)		0.02	0.10	14.8*(4.3~26.3)

Abbreviations: ICD-10, International Classification of Diseases 10th revision; ASMR, age-standardized mortality rate; AAPC: average annual percentage change; CI: confidence interval

*: statistically significance

### Projections of gynecological cancers in China

**Fig. 1:**
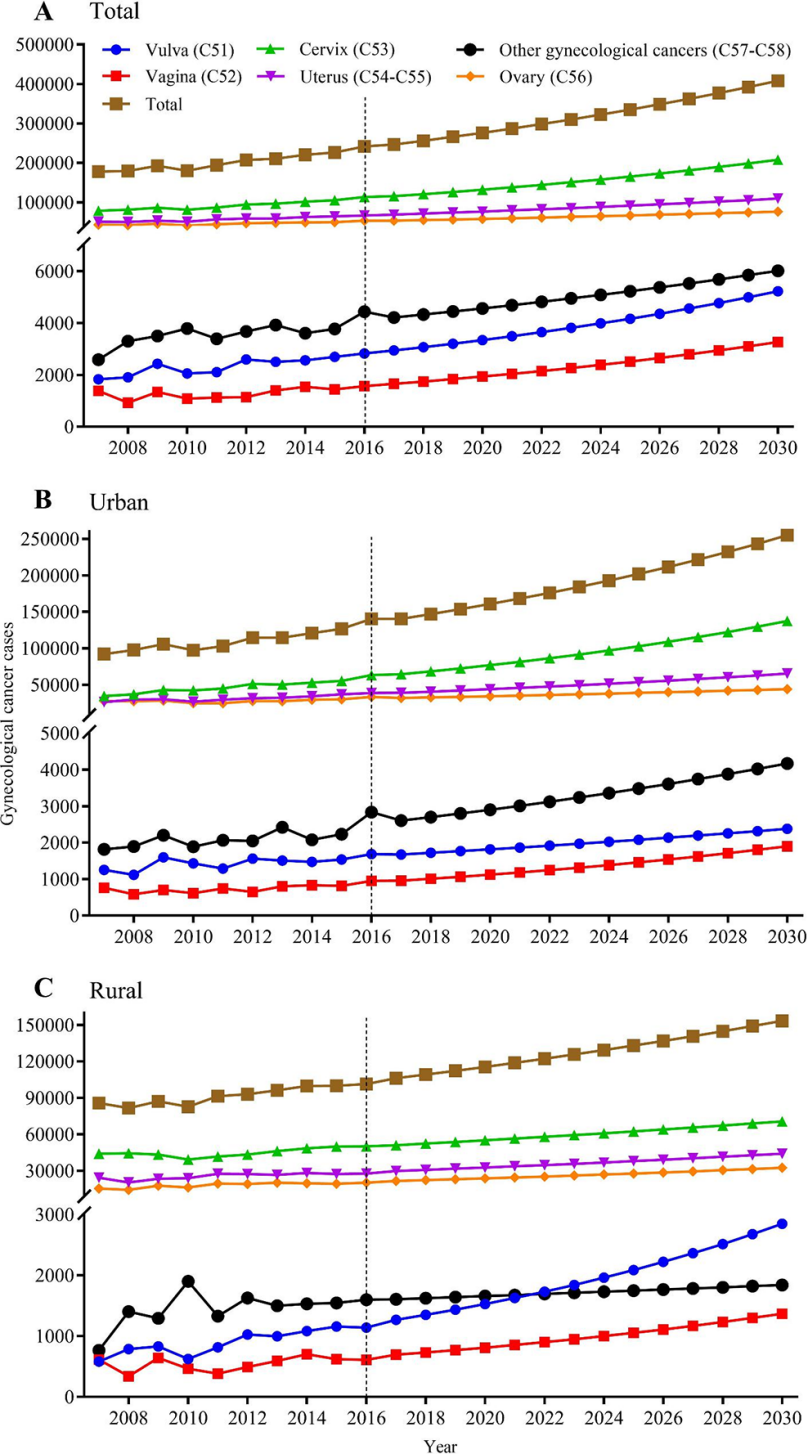
Projected gynecological cancer cases in China, 2017–2030, in total population (A), in urban females (B), and in rural females (C). The figure contains three panels. The first (A) is a line graph of different projected gynecological cancer cases among the total population, with the case on the y-axis and year on the x-axis. The second (B) is a line graph of different projected gynecological cancer cases among urban females, with the case on the y-axis and year on the x-axis. The third (C) is a line graph of different projected gynecological cancer cases among rural females, with the case on the y-axis and year on the x-axis. The legend on the top displays the line shape and color of each gynecological cancer and the legend is commonly applied to all three panels in this figure. For the three panels in this figure, the cancer cases during 2007–2016 is calculated by multiplying age-specific rates with the estimated population size, while the cancer cases during 2017–2030 is projected using grey prediction model GM (1,1). More details on the data source and statistical methods of estimation and prediction can be referred to Materials and Methods section.

Figure 1, Additional File Figure S9 and Supplementary Tables S7 to S8 showed the projection of gynecological cancer cases and incidence from 2017 to 2030. The total projected cancer cases are changing from 246,581 to 408,314, and the age-standardized incidence rate is rising from 24.99 to 34.87 per 100,000 persons. In 2030, 51.0% of projected cancer cases are cervical cancer (208,054), follow by uterine (109,314), ovarian (76,433), other gynecological (6,015), vulva (5,231), and vaginal (3,271). The total gynecological cancer cases in the next decade are projected growing trend, cervical, vulva and vaginal cancers showed evident upward trend, uterine cancer are slightly increasing, while ovarian cancer remain unchanged. The projected ASIR of total gynecological cancers is increasing, with slowly growing for cervical, vulva, and vaginal cancer while the others are maintaining. Urban and rural were predicted similar trends except that vulva cancer cases and incidence are increasing higher in rural areas.

**Fig. 2:**
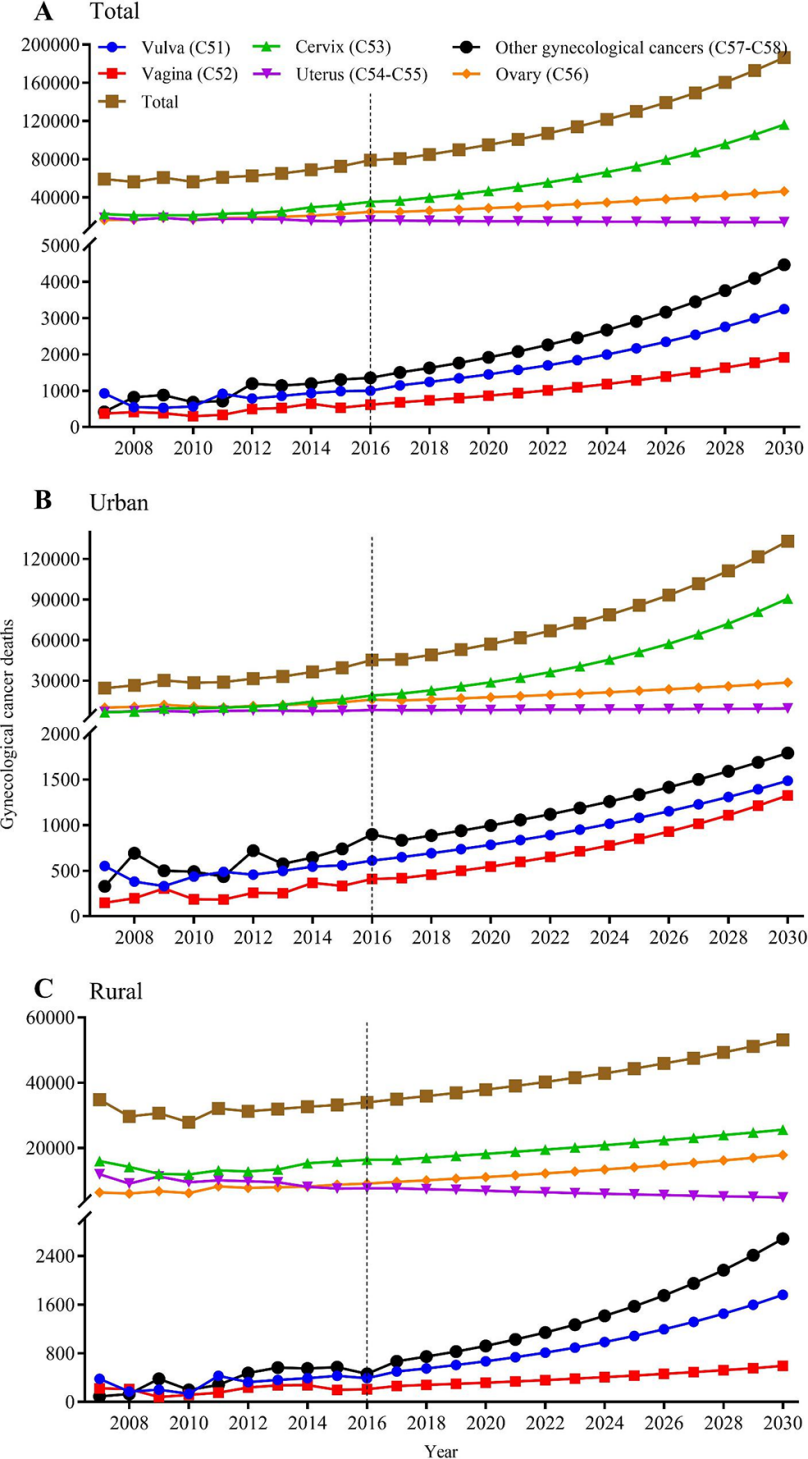
Projected gynecological cancer deaths in China, 2017–2030, in total population (A), in urban females (B), and in rural females (C). The figure contains three panels. The first (A) is a line graph of different projected gynecological cancer deaths among the total population, with the death on the y-axis and year on the x-axis. The second (B) is a line graph of different projected gynecological cancer deaths among urban females, with the death on the y-axis and year on the x-axis. The third (C) is a line graph of different projected gynecological cancer deaths among rural females, with the death on the y-axis and year on the x-axis. The legend on the top displays the line shape and color of each gynecological cancer and the legend is commonly applied to all three panels in this figure. For the three panels in this figure, the cancer deaths during 2007–2016 is calculated by multiplying age-specific rates with the estimated population size, while the cancer deaths during 2017–2030 is projected using grey prediction model GM (1,1). More details on the data source and statistical methods of estimation and prediction can be referred to Materials and Methods section.

Figure 2, Additional File Figure S10 and Supplementary Tables S7 to S8 presented the projection of gynecological cancer deaths and mortality during 2017–2030. The projected total cancer deaths are rising from 80,661 to 186,360, and the age-standardized mortality rate is increasing from 7.70 to 12.35 per 100,000 persons. In 2030, 62.4% of predicted cancer deaths are cervical cancer (116,315), follow by ovarian (46,318), uterine (14,093), other gynecological (4,471), vulva (3,247), and vaginal cancer (1,916). All cancer deaths are rising in the future except the uterine cancer, urban and rural showed the similar trends but the vaginal cancer deaths are growing higher in urban. The projected cancer mortality trends are similar with the deaths, but vulva and other gynecological cancer mortality are growing faster in rural areas.

## Discussion

Gynecological cancers were responsible for 241,800 cancer cases and 79,179 cancer deaths in China in 2016, accounting for 5.9% and 3.3% of the nationwide burden of total new cancer cases (4,064,000) and cancer deaths (2,413,500) respectively [17]. Cervical, uterine, and ovarian cancer ranked the first three burden. All the gynecological cancers were identified increasing trend in the past and were predicted elevation in the future on varying degrees, and the most evident growing was observed on cervical and vulva cancer. Ovarian cancer death and uterine cancer cases is also continuous climbing.

Cervical cancer burden is high in China with 113,256 new cancer cases and 35,353 cancer deaths occurred annually, it was increased by average 4.1% on cases and 5.1% on deaths per year during the past decades, and the cancer burden were projected continually increasing in the next decade. Differently, some high-income countries revealed that cervical cancer has been observed decreasing in the past few decades due to organized screening and HPV vaccination [18, 19]. The intensively increasing disease burden of cervical cancer highlighted the urgency of comprehensive control strategies in China, including HPV vaccination program for girls aged 9–14 and screening for women aged 35–64. China is now highly acting at accelerating cervical cancer elimination including expanding HPV vaccination and screening coverage. The temporal trends of cervical cancer in the future might be influenced.

The elevation for ovarian cancer is higher on deaths (AAPC: 4.5%) than that of on cases (AAPC: 2.4%) during 2007–2016, and the cancer deaths is predicted evident rising but cases is unchangeable in the next decade in China. Growing incidence and mortality of ovarian cancer were also reported in developed areas in China [20, 21]. Decreasing trend of ovarian cancer was observed globally, but the disease burden has been still increasing in some lower-income countries and among younger females in some countries [5]. Screening for ovarian cancer is not currently recommended in the general population, and no feasible screening method as well. Large scale randomized clinic trials have revealed that neither transvaginal ultrasound nor cancer antigen 125 (CA-125) testing could decrease ovarian cancer mortality [22]. Most ovarian cancers are diagnosed in advanced clinical stages with poor prospects of long-term survival, early detection in high-risk women is highly recommended. Optimal biomarker including the CA-125, Human epididymal protein 4 (HE-4), BRCA gene, and other new biomarkers for early detection needs to be explored. Multidisciplinary treatment involving surgery, radiotherapy and chemotherapy are important in reducing mortality from ovarian cancer.

Uterine cancer cases and incidence are continuously climbing, while the cancer deaths and mortality were declined during 2007–2030 in China. It was similar with the global trends that the age-standardized incidence is increased and the age-standardized mortality is decreased between 1990 and 2017 [23]. Uterine cancer incidence rates were observed increased especially in those countries with rapid socioeconomic transitions [24]. Both incidence and mortality of uterine cancer were also reported growing in developed areas in China [20, 21]. The elevation of uterine cancer is highly related to high body mass index, waist-to-hip ratio [23, 25], continuing increases in obesity, and decreases in fertility [24]. The declined fertility rates in some Asian countries putting more women at greater risk of uterine cancer [24]. As a postmenopausal disease, uterine cancer will become a more important public health problem in future years. Nevertheless, no feasible screening method for uterine cancer, and the potential benefit of screening among asymptomatic women still lack of evidence. Women with Lynch Syndrome have a 12–47% lifetime risk of developing endometrial cancer and were recommend annual surveillance using transvaginal ultrasound and endometrial biopsy from the age of 30–35 years or 40 years [26].

The rare cancers including vulva, vaginal, and other gynecological cancers are increasing in the past and future in China. Similarly, vulva and vaginal cancer are also reported consistently growing globally [4]. Screening for these rare cancers is not cost-benefit at a population level, and no optimal screening strategies yet. Around 78% of vaginal and 24% of vulva cancers are attributable to HPV infection [6] that would been prevented by HPV vaccination. The prevention of vulva and vaginal cancer would be benefit from cervical cancer control in China.

In China, a rapidly increasing trend was observed among individuals older than 65 years (average annual percent change (AAPC): 3.67), and a slow rising trend was observed among individuals aged 15–64 years (AAPC: 1.10) during 2000 to 2019 [27]. With the aging of population, late marriage, fertility decline, and increase of unhealthy lifestyles, the burden of gynecological cancer in China is likely to be increased rapidly in the future. The projections on temporal changes of gynecological cancers are urgently needed.

This study has estimated the burden, temporal trend, and prediction of gynecological cancer with cases, incidence, deaths, and mortality by age and geographical areas in past and next decade in China. Our study provides a comprehensive nationwide profile on the burden of gynecological cancer at a population level, supplies fundamental data and scientific evidences for policy making on gynecological cancer control and prevention. However, there is a limitation of this study, the predicted cancer burden in this study is based on a statistical projection model and no potential advances in the prevention, diagnosis, or therapy that may dramatically impact future incidence rates were taken into accounts, which may negatively impact the precision of estimation of the actual cancer burden and the predicted burden.

## Conclusion

Gynecological cancer burden in China was substantial, with cervical, uterine, and ovarian cancer contributed the vast majority new cancer cases and deaths. Significant increasing trend of gynecological cancer burden was observed in the past and future, and the most evident increases were cervical and vulva cancers. Ovarian cancer death and mortality, as well as uterine cancer cases and incidence is also continuous growing. With the aging of population and other increased risk factors, the burden of gynecological cancers in China is likely to be grew rapidly in the future, comprehensive gynecological cancer control should be concerned. Further evaluation for gynecological cancer burden with a scenario-based forecast model considering the possible changes are expected to provide a more precise and tailored cancer burden estimation in China.

## Electronic supplementary material

Below is the link to the electronic supplementary material.

Additional File 1: Figure s1-s10

Additional File 2: Table s1-s8

## Data Availability

The datasets analyzed during the current study are available in the Chinese Cancer Registry Annual Report that published by the National Central Cancer Registry of China (NCCR), and in the China Population & Employment Statistics Yearbook that released by National Bureau of Statistics of China. The data and images generated in this study are available upon request from the corresponding author.

## Declarations

### Enhancing the quality and transparency of health research (EQUATOR) network guidelines

This study followed the EQUATOR network guidelines.

## Abbreviations

HPV: human papillomavirus
ASIR: age-standardized incidence rate
ASMR: age-standardized mortality rate. AAPC:average annual percentage change
