# Supplementary material for: Temporal trends and projections of gynecological cancers in China, 2007–2030
Source: BMC Womens Health. 2023 Jun 30;23:346. doi: 10.1186/s12905-023-02384-2 (PMC10311708; doi:10.1186/s12905-023-02384-2)
Supplement: Supplementary file 1 — Additional File 1: Figure s1-s10 [file 12905_2023_2384_MOESM1_ESM.docx]

**Title: Temporal trends and projections of gynecological cancers in China, 2007-2030**

**Supplementary Figures**


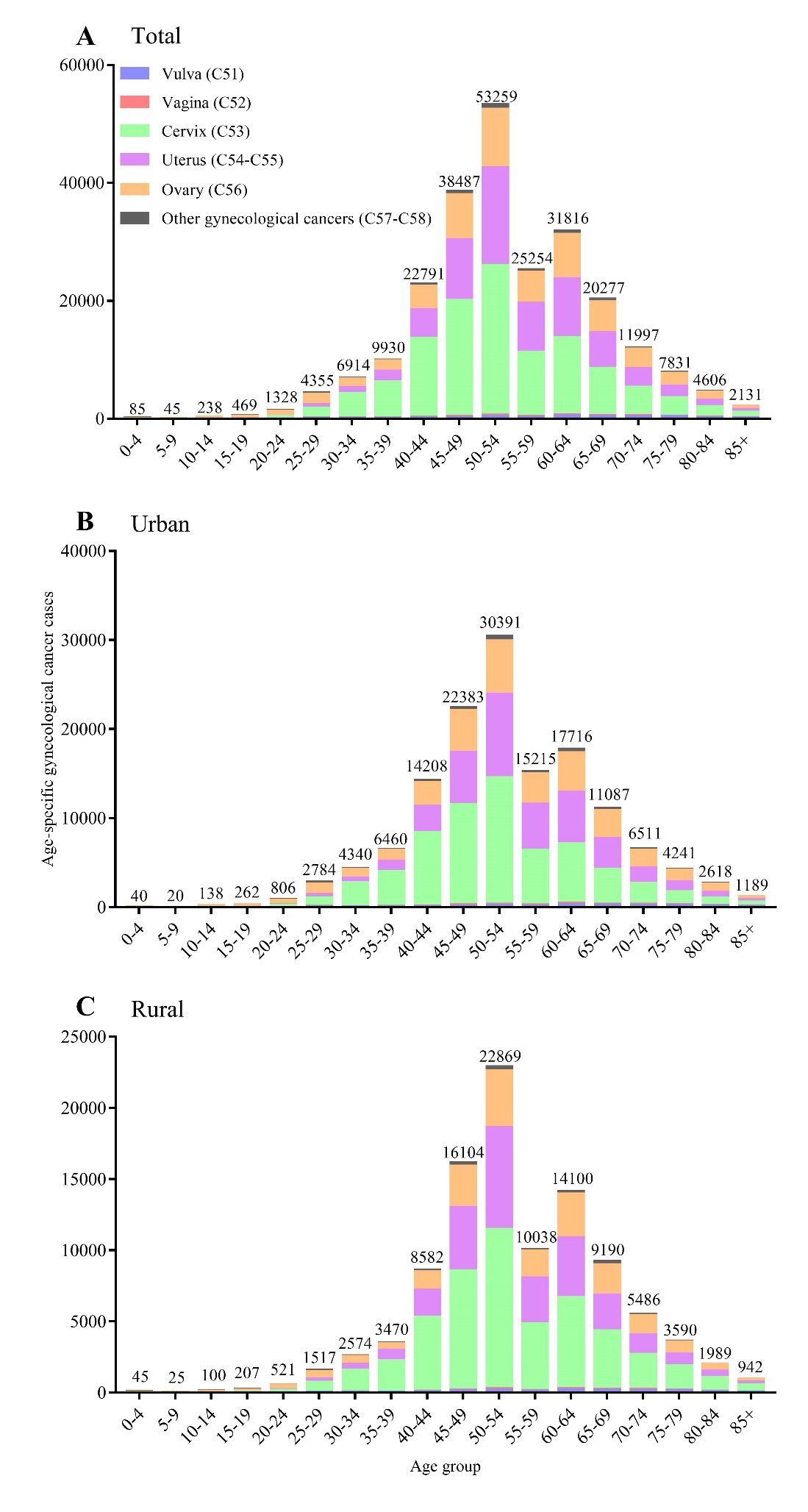


Figure S1. Age-specific gynecological cancer cases in China, 2016, in total population (A), in urban females (B), and in rural females (C). The figure contains three panels. The first (A) is a bar chart of different gynecological cancer cases among the total population, with the case on the y axis and age group on the x axis. The second (B) is a bar chart of different gynecological cancer cases among urban females, with the case on the y axis and age group on the x axis. The third (C) is a bar chart of different gynecological cancer cases among rural females, with the case on the y axis and age group on the x axis. The legend on the top left corner displays each gynecological cancer and the legend is commonly applied to all three panels in this figure.


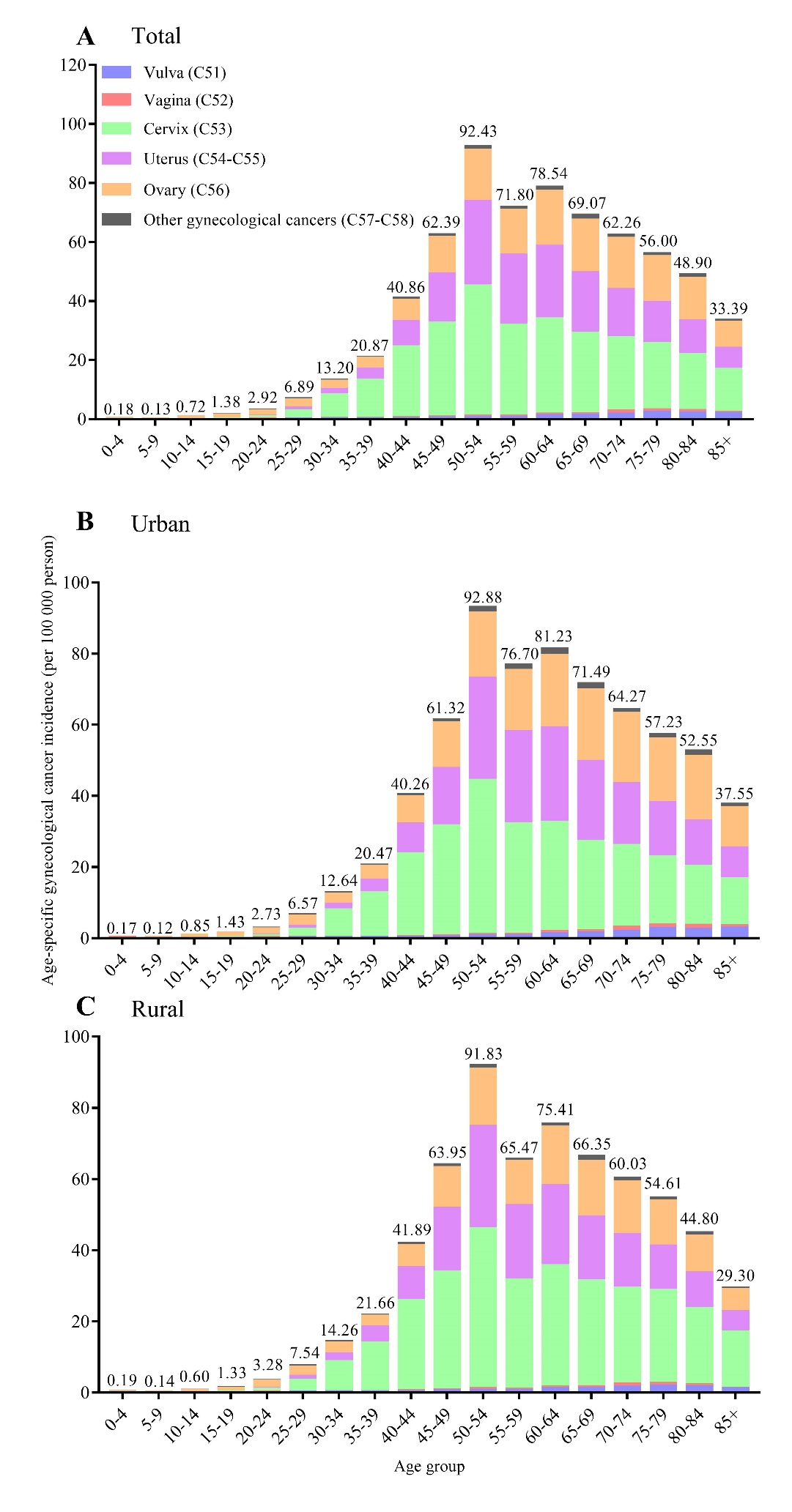


Figure S2. Age-specific gynecological cancer incidence in China, 2016, in total population (A), in urban females (B), and in rural females (C). The figure contains three panels. The first (A) is a bar chart of different gynecological cancer incidence among the total population, with the incidence on the y axis and age group on the x axis. The second (B) is a bar chart of different gynecological cancer incidence among urban females, with the incidence on the y axis and age group on the x axis. The third (C) is a bar chart of different gynecological cancer incidence among rural females, with the incidence on the y axis and age group on the x axis. The legend on the top left corner displays each gynecological cancer and the legend is commonly applied to all three panels in this figure.


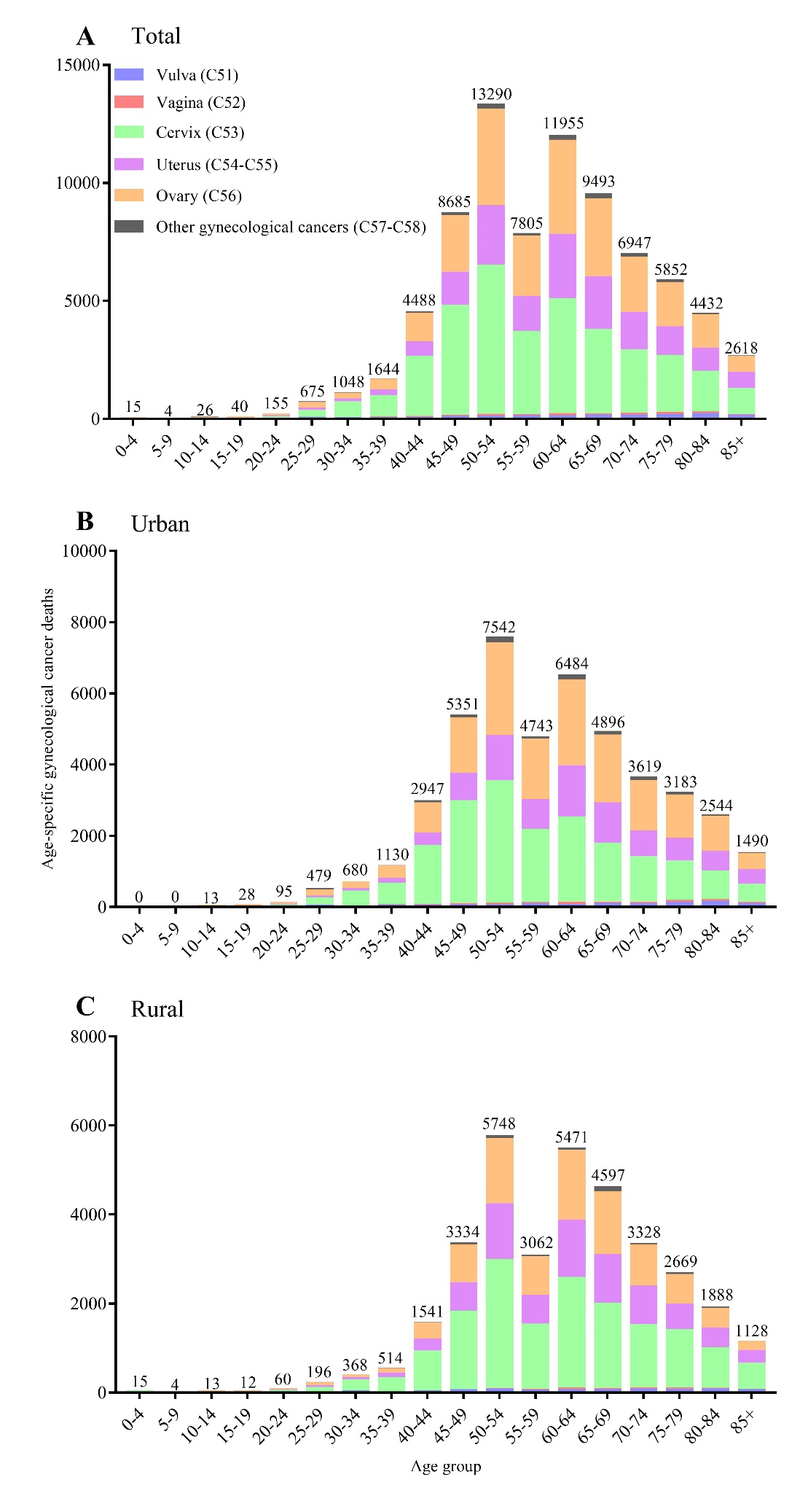


Figure S3. Age-specific gynecological cancer deaths in China, 2016, in total population (A), in urban females (B), and in rural females (C). The figure contains three panels. The first (A) is a bar chart of different gynecological cancer deaths among the total population, with the death on the y axis and age group on the x axis. The second (B) is a bar chart of different gynecological cancer deaths among urban females, with the death on the y axis and age group on the x axis. The third (C) is a bar chart of different gynecological cancer deaths among rural females, with the death on the y axis and age group on the x axis. The legend on the top left corner displays each gynecological cancer and the legend is commonly applied to all three panels in this figure.


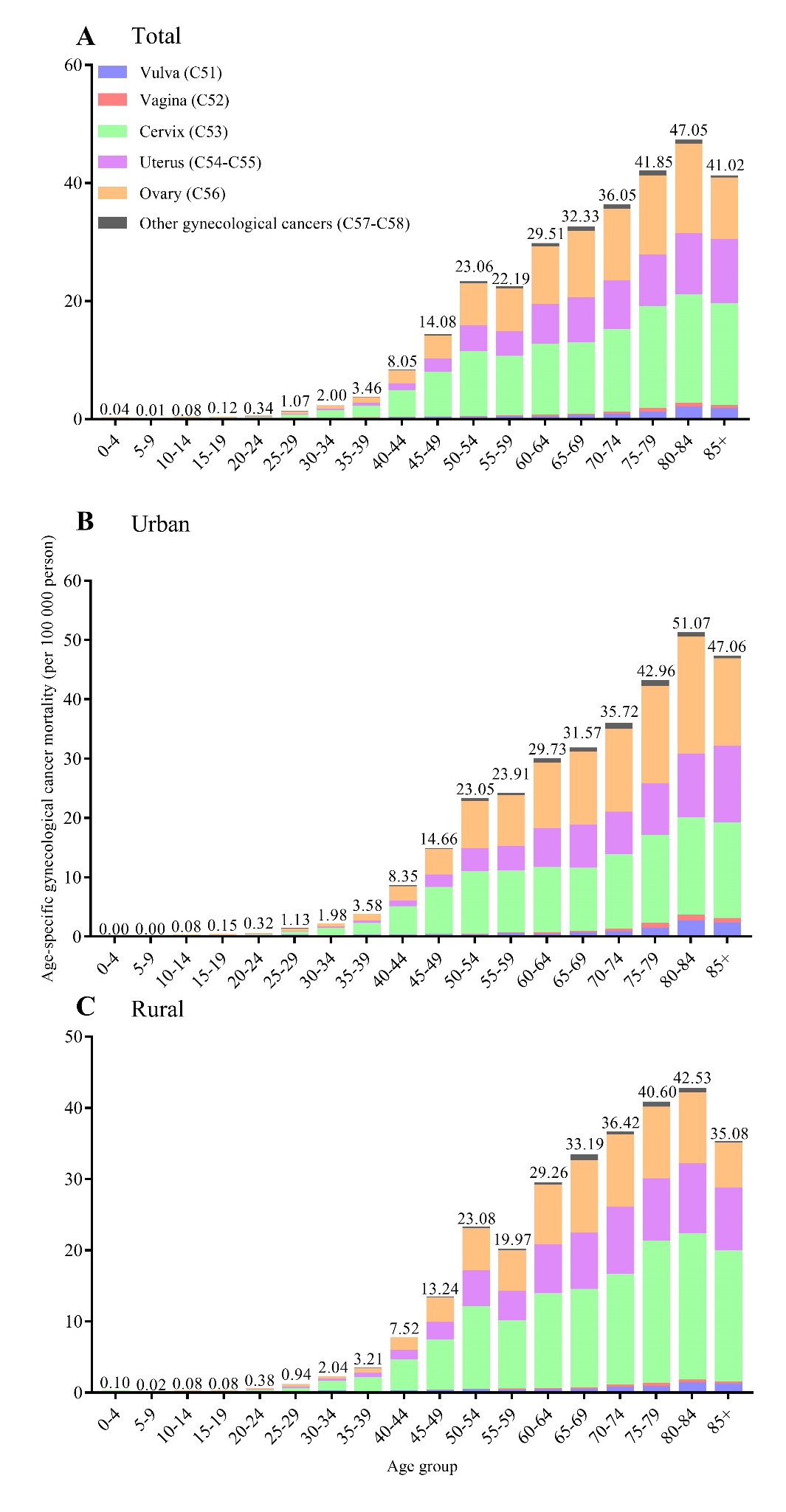


Figure S4. Age-specific gynecological cancer mortality in China, 2016, in total population (A), in urban females (B), and in rural females (C). The figure contains three panels. The first (A) is a bar chart of different gynecological cancer mortality among the total population, with the mortality on the y axis and age group on the x axis. The second (B) is a bar chart of different gynecological cancer mortality among urban females, with the mortality on the y axis and age group on the x axis. The third (C) is a bar chart of different gynecological cancer mortality among rural females, with the mortality on the y axis and age group on the x axis. The legend on the top left corner displays each gynecological cancer and the legend is commonly applied to all three panels in this figure.


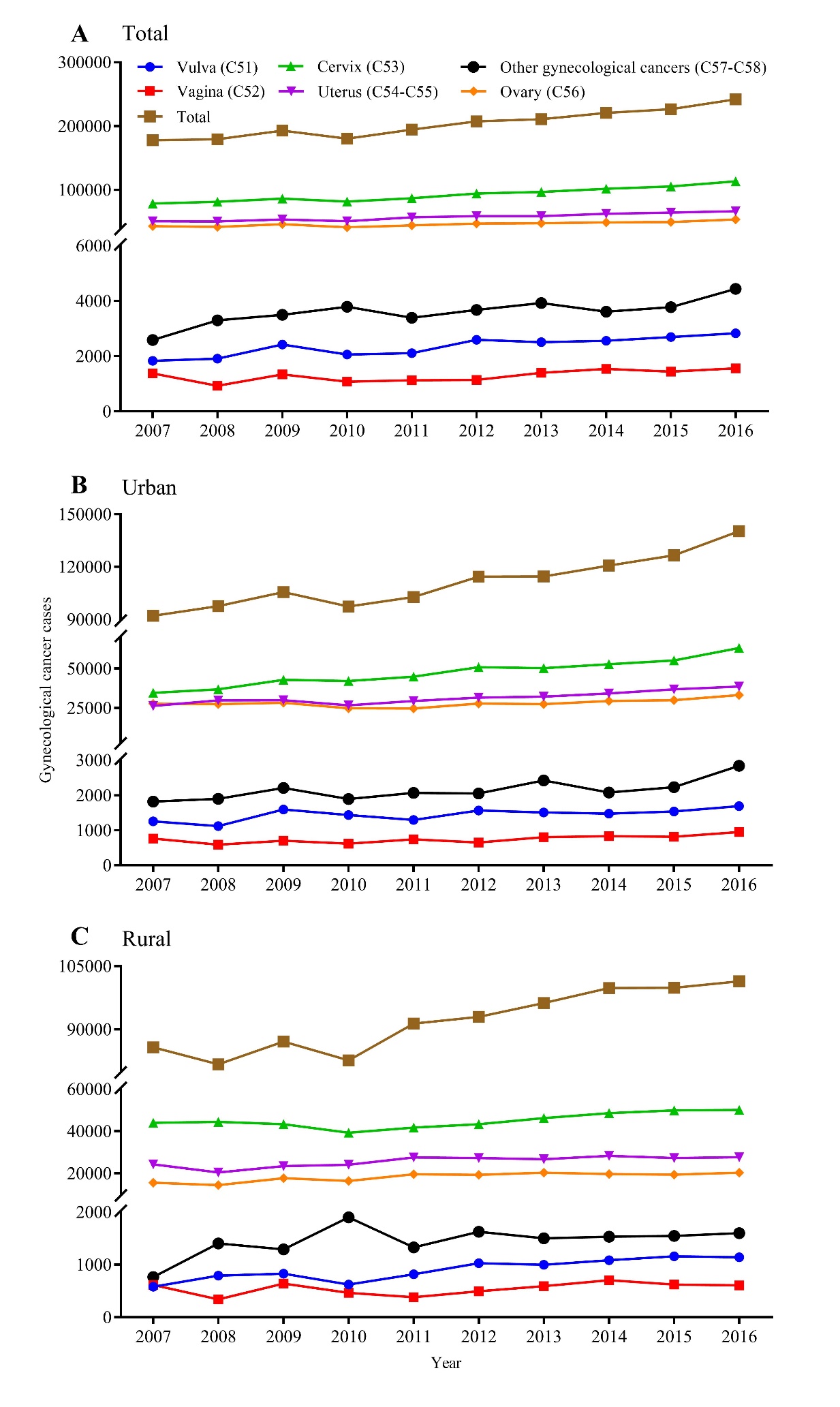


Figure S5. Trends of gynecological cancer cases in China, 2007-2016, in total population (A), in urban females (B), and in rural females (C). The figure contains three panels. The first (A) is a line graph of different gynecological cancer cases among the total population, with the case on the y axis and year on the x axis. The second (B) is a line graph of different gynecological cancer cases among urban females, with the case on the y axis and year on the x axis. The third (C) is a line graph of different gynecological cancer cases among rural females, with the case on the y axis and year on the x axis. The legend on the top displays the line shape and color of each gynecological cancer and the legend is commonly applied to all three panels in this figure.


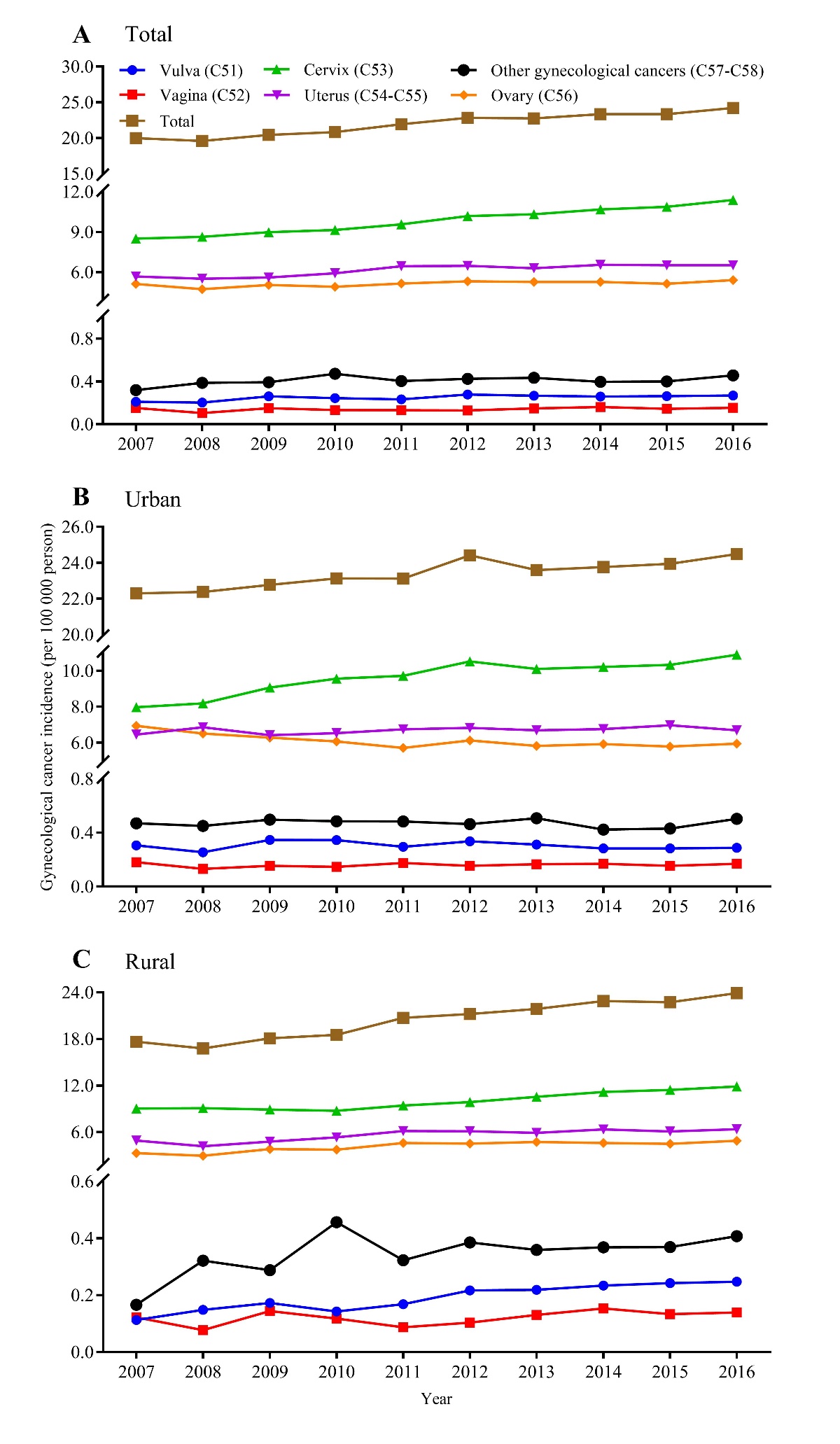


Figure S6. Trends of gynecological cancer incidence in China, 2007-2016, in total population (A), in urban females (B), and in rural females (C). The figure contains three panels. The first (A) is a line graph of different gynecological cancer incidence among the total population, with the incidence on the y axis and year on the x axis. The second (B) is a line graph of different gynecological cancer incidence among urban females, with the incidence on the y axis and year on the x axis. The third (C) is a line graph of different gynecological cancer incidence among rural females, with the incidence on the y axis and year on the x axis. The legend on the top displays the line shape and color of each gynecological cancer and the legend is commonly applied to all three panels in this figure.


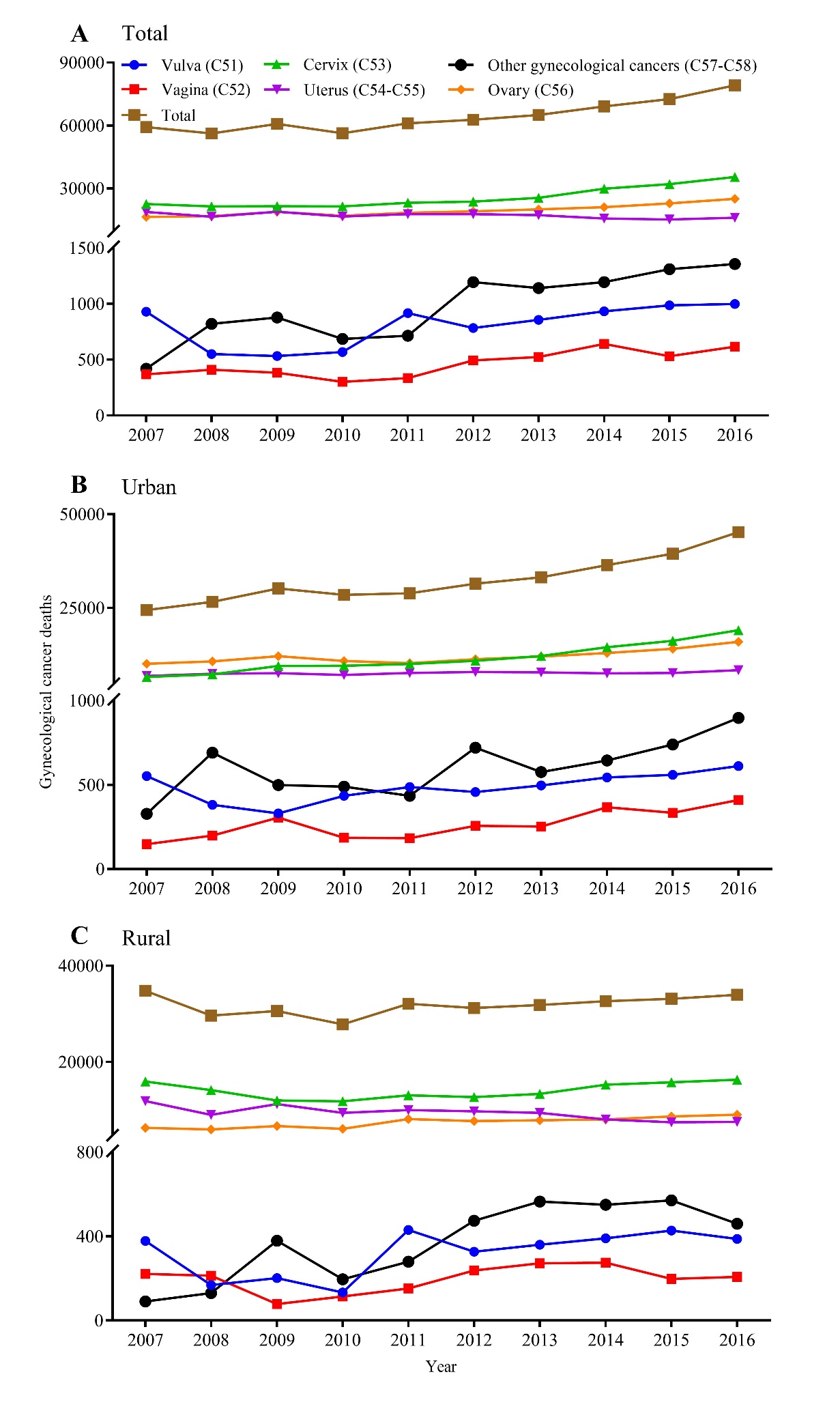


Figure S7. Trends of gynecological cancer deaths in China, 2007-2016, in total population (A), in urban females (B), and in rural females (C). The figure contains three panels. The first (A) is a line graph of different gynecological cancer deaths among the total population, with the death on the y axis and year on the x axis. The second (B) is a line graph of different gynecological cancer deaths among urban females, with the death on the y axis and year on the x axis. The third (C) is a line graph of different gynecological cancer deaths among rural females, with the death on the y axis and year on the x axis. The legend on the top displays the line shape and color of each gynecological cancer and the legend is commonly applied to all three panels in this figure.


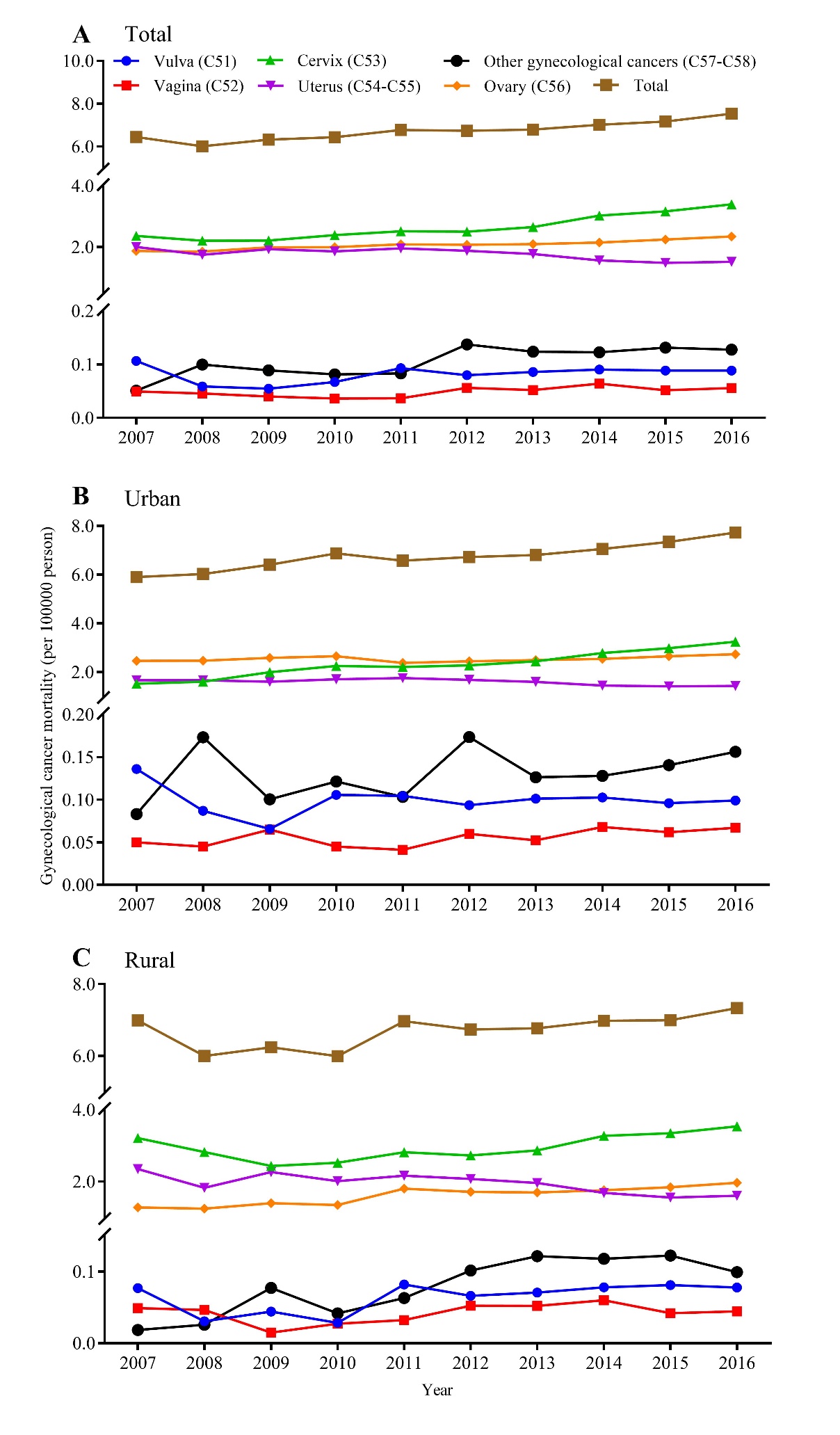


Figure S8. Trends of gynecological cancer mortality in China, 2007-2016, in total population (A), in urban females (B), and in rural females (C). The figure contains three panels. The first (A) is a line graph of different gynecological cancer mortality among the total population, with the mortality on the y axis and year on the x axis. The second (B) is a line graph of different gynecological cancer mortality among urban females, with the mortality on the y axis and year on the x axis. The third (C) is a line graph of different gynecological cancer mortality among rural females, with the mortality on the y axis and year on the x axis. The legend on the top displays the line shape and color of each gynecological cancer and the legend is commonly applied to all three panels in this figure.


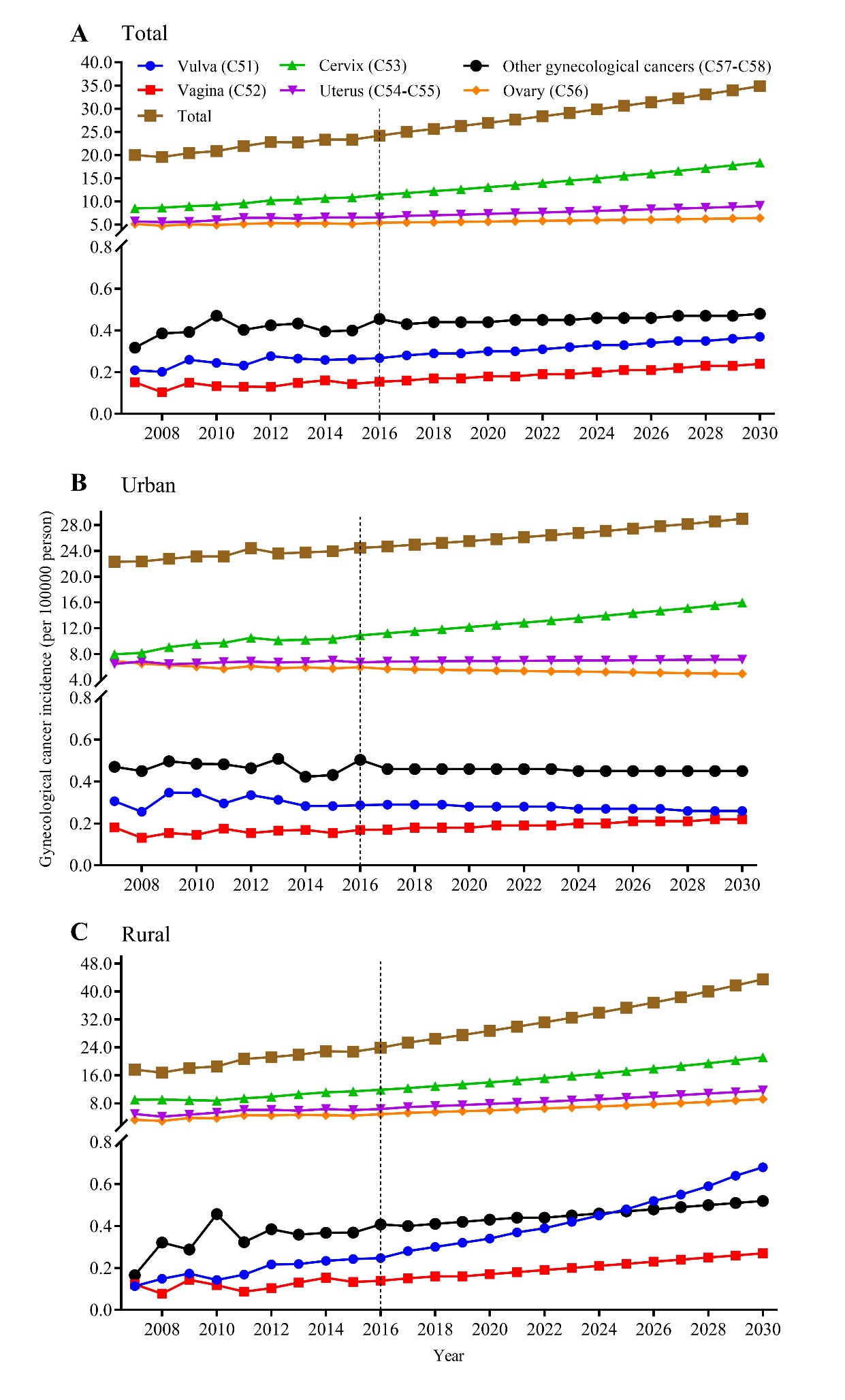


Figure S9. Projected gynecological cancer incidence in China, 2017-2030, in total population (A), in urban females (B), and in rural females (C). The figure contains three panels. The first (A) is a line graph of different projected gynecological cancer incidence among the total population, with the incidence on the y-axis and year on the x-axis. The second (B) is a line graph of different projected gynecological cancer incidence among urban females, with the incidence on the y-axis and year on the x-axis. The third (C) is a line graph of different projected gynecological cancer incidence among rural females, with the incidence on the y-axis and year on the x-axis. The legend on the top displays the line shape and color of each gynecological cancer and the legend is commonly applied to all three panels in this figure. For the three panels in this figure, the cancer incidence during 2007–2016 is compounded by the estimated cases and total population, while the cancer incidence during 2017–2030 is projected using grey prediction model GM (1,1). More details on the data source and statistical methods of estimation and prediction can be referred to Materials and Methods section.


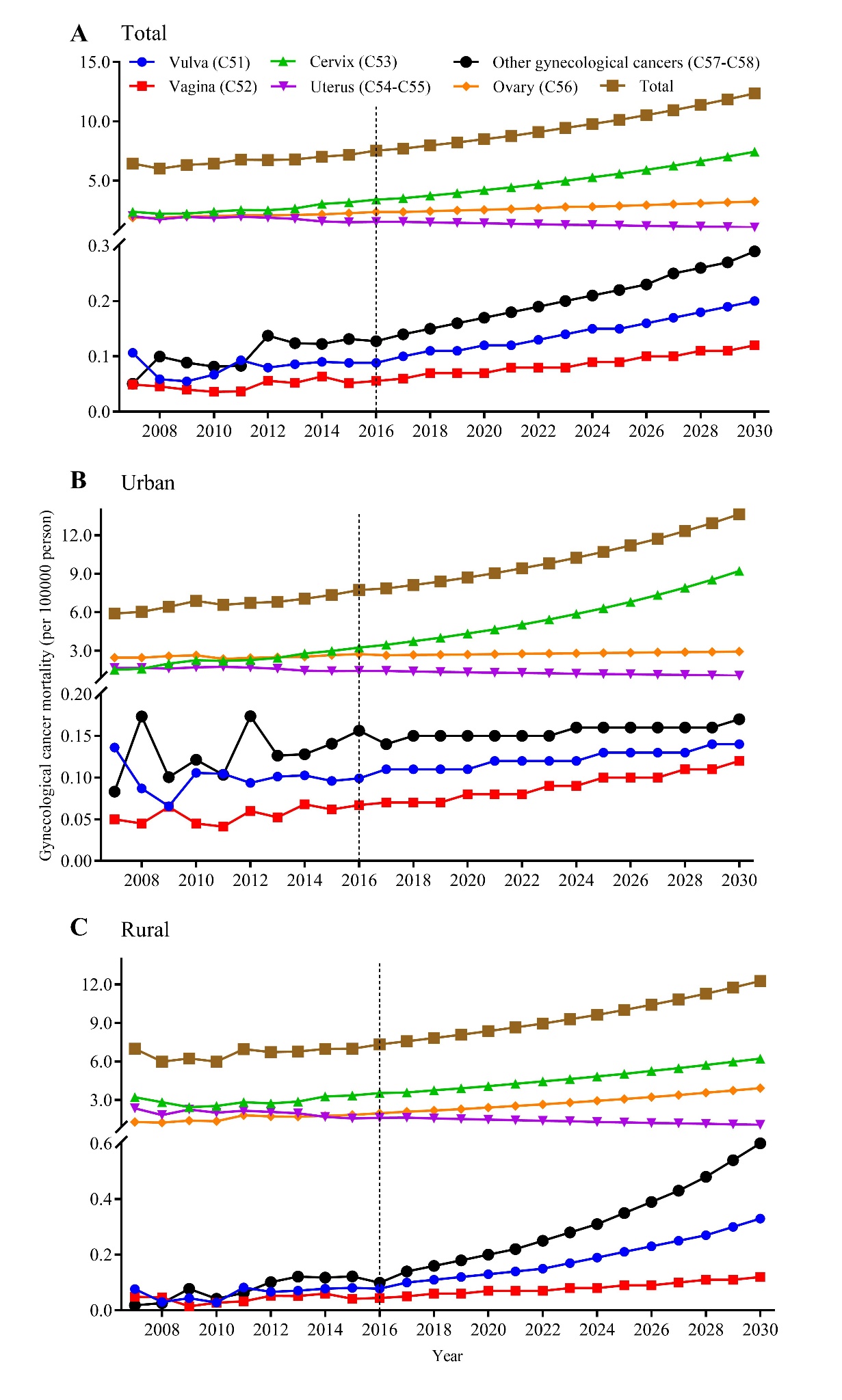


Figure S10. Projected gynecological cancer mortality in China, 2017-2030, in total population (A), in urban females (B), and in rural females (C). The figure contains three panels. The first (A) is a line graph of different projected gynecological cancer mortality among the total population, with the mortality on the y-axis and year on the x-axis. The second (B) is a line graph of different projected gynecological cancer mortality among urban females, with the mortality on the y-axis and year on the x-axis. The third (C) is a line graph of different projected gynecological cancer mortality among rural females, with the mortality on the y-axis and year on the x-axis. The legend on the top displays the line shape and color of each gynecological cancer and the legend is commonly applied to all three panels in this figure. For the three panels in this figure, the cancer mortality during 2007–2016 is compounded by the estimated deaths and total population, while the cancer mortality during 2017–2030 is projected using grey prediction model GM (1,1). More details on the data source and statistical methods of estimation and prediction can be referred to Materials and Methods section.
