# Supplementary material for: Temporal trends and projections of gynecological cancers in China, 2007–2030
Source: BMC Womens Health. 2023 Jun 30;23:346. doi: 10.1186/s12905-023-02384-2 (PMC10311708; doi:10.1186/s12905-023-02384-2)
Supplement: Supplementary file 2 — Additional File 2: Table s1-s8 [file 12905_2023_2384_MOESM2_ESM.docx]

**Title: Temporal trends and projections of gynecological cancers in China, 2007-2030**

**Supplementary Tables**

| **Table S1 Trends analysis of cases, incidence, deaths, and mortality of vulva cancer in China, by age, 2007-2016** | | | | | | | | | | | | |
| --- | --- | --- | --- | --- | --- | --- | --- | --- | --- | --- | --- | --- |
|  | **Cancer cases** | | | **Incidence** | | | **Cancer deaths** | | | **Mortality** | | |
| **Age group** | **2007** | **2016** | **AAPC (95% CI)** | **2007** | **2016** | **AAPC (95% CI)** | **2007** | **2016** | **AAPC (95% CI)** | **2007** | **2016** | **AAPC (95% CI)** |
| **Total** | 1883 | 2827 | 4.4*(2.5～6.4) | 0.21 | 0.26 | 2.5*(0.6～4.5) | 930 | 999 | 4.3(-0.8～9.6) | 0.11 | 0.09 | 1.3(-3.7～6.6) |
| 15-19 | 16 | 0 | - | 0.03 | 0.00 | - | 0 | 0 | - | 0.00 | 0.00 | - |
| 20-24 | 12 | 18 | - | 0.03 | 0.04 | - | 0 | 0 | - | 0.00 | 0.00 | - |
| 25-29 | 40 | 55 | 3.7(-2.6～10.5) | 0.09 | 0.09 | -1(-7.3～5.8) | 14 | 17 | - | 0.03 | 0.03 | - |
| 30-34 | 46 | 51 | - | 0.09 | 0.10 | - | 0 | 14 | - | 0.00 | 0.03 | - |
| 35-39 | 81 | 59 | -9.0*(-14.9～-2.7) | 0.13 | 0.12 | -5.4(-11.3～0.9) | 0 | 13 | - | 0.00 | 0.03 | - |
| 40-44 | 161 | 110 | -5.2(-11.9～1.9) | 0.25 | 0.20 | -3.9(-10.7～3.3) | 89 | 20 | -12.6*(-19.2～-5.4) | 0.14 | 0.04 | -11.4*(-18.2～-4.0) |
| 45-49 | 22 | 223 | 8.3(-2.4～20.3) | 0.05 | 0.36 | 5.5(-4.2～16.1) | 22 | 64 | 14.6*(2.2～28.4) | 0.05 | 0.10 | 11.1(-0.7～24.5) |
| 50-54 | 274 | 336 | 4.5(-1.4～10.8) | 0.54 | 0.58 | 3.2(-0.4～7) | 12 | 73 | - | 0.02 | 0.13 | - |
| 55-59 | 96 | 203 | 3.9(-1.9～10.1) | 0.24 | 0.58 | 5.9*(0.9～11.3) | 67 | 80 | - | 0.17 | 0.23 | - |
| 60-64 | 168 | 461 | 6.1*(0.2～12.3) | 0.60 | 1.14 | 1.7(-3.8～7.4) | 63 | 77 | 10.6*(0.9～21.2) | 0.23 | 0.19 | 5.7(-3.6～16.0) |
| 65-69 | 198 | 365 | 8.3*(1.5～15.7) | 0.91 | 1.24 | 4.4(-2.5～11.9) | 187 | 111 | -2.3(-10.9～7.1) | 0.86 | 0.38 | -5.2(-13.1～3.4) |
| 70-74 | 399 | 322 | -0.6(-5.6～4.8) | 2.13 | 1.67 | -0.5(-5.2～4.3) | 142 | 114 | 2.6(-6.6～12.8) | 0.76 | 0.59 | 2.6(-6.6～12.7) |
| 75-79 | 142 | 314 | 8.8*(2.5～15.5) | 1.17 | 2.24 | 7.3*(1.1～14) | 76 | 135 | 6.8(-1.6～15.9) | 0.63 | 0.96 | 5.4(-2.6～14.1) |
| 80-84 | 120 | 189 | 3.1(-2.1～8.5) | 1.70 | 2.01 | -0.3(-5.2～4.9) | 127 | 174 | 7.5(-0.2～15.8) | 1.80 | 1.85 | 4(-3.5～12.1) |
| 85+ | 58 | 119 | 5.0*(0.6～9.6) | 1.42 | 1.86 | 0(-4.2～4.5) | 131 | 99 | -0.8(-7～5.7) | 3.23 | 1.56 | -5.6(-11.6～0.9) |

Abbreviations: AAPC: average annual percentage change; CI: confidence interval.

*: statistically significance.

| **Table S2 Trends analysis of cases, incidence, deaths, and mortality of vaginal cancer in China, by age, 2007-2016** | | | | | | | | | | | | |
| --- | --- | --- | --- | --- | --- | --- | --- | --- | --- | --- | --- | --- |
|  | **Cancer cases** | | | **Incidence** | | | **Cancer deaths** | | | **Mortality** | | |
| **Age group** | **2007** | **2016** | **AAPC (95% CI)** | **2007** | **2016** | **AAPC (95% CI)** | **2007** | **2016** | **AAPC (95% CI)** | **2007** | **2016** | **AAPC (95% CI)** |
| Total | 1378 | 1561 | 3.2(-0.3～6.8) | 0.15 | 0.15 | 1.6(-1.3～4.6) | 370 | 616 | 7.0*(2.8～11.3) | 0.05 | 0.06 | 3.5(-0.6～7.8) |
| 15-19 | 0 | 0 | - | 0.00 | 0.00 | - | 0 | 0 | - | 0.00 | 0.00 | - |
| 20-24 | 0 | 14 | - | 0.00 | 0.03 | - | 0 | 0 | - | 0.00 | 0.00 | - |
| 25-29 | 0 | 23 | - | 0.00 | 0.04 | - | 0 | 0 | - | 0.00 | 0.00 | - |
| 30-34 | 48 | 25 | -14.7*(-26.4～-1.2) | 0.10 | 0.05 | -15.4*(-27～-2) | 0 | 0 | - | 0.00 | 0.00 | - |
| 35-39 | 48 | 35 | -9(-21.7～5.7) | 0.07 | 0.07 | -5.4(-18.4～9.6) | 0 | 16 | - | 0.00 | 0.03 | - |
| 40-44 | 161 | 97 | -4.5*(-8.7～-0.2) | 0.25 | 0.17 | -3.2(-7.3～1.2) | 0 | 27 | - | 0.00 | 0.05 | - |
| 45-49 | 109 | 190 | 7.6*(3.1～12.4) | 0.26 | 0.31 | 4.1(-0.4～8.8) | 42 | 34 | -0.1(-11.1～12.4) | 0.10 | 0.06 | -3.7(-14.5～8.4) |
| 50-54 | 150 | 222 | 9.8*(1.5～18.8) | 0.29 | 0.39 | 8.2*(0.4～16.6) | 12 | 63 | 11.4(-1～25.5) | 0.02 | 0.11 | 8.1(-4.7～22.6) |
| 55-59 | 449 | 135 | -9.3*(-16.8～-1.1) | 1.12 | 0.39 | -8.4(-16.7～0.7) | 89 | 54 | -5.1(-14.4～5.2) | 0.22 | 0.15 | -3.6(-13.3～7.1) |
| 60-64 | 159 | 212 | 4.1(-3.8～12.6) | 0.57 | 0.52 | -0.3(-7.4～7.5) | 109 | 97 | 0.6(-8.1～10.2) | 0.39 | 0.24 | -3.5(-11.6～5.3) |
| 65-69 | 35 | 169 | 6.7(-0.6～14.6) | 0.16 | 0.57 | 2.8(-4.2～10.4) | 0 | 57 | - | 0.00 | 0.20 | - |
| 70-74 | 163 | 201 | 2.9(-3.8～10.1) | 0.87 | 1.04 | 2.7(-3.5～9.3) | 81 | 73 | 3.1(-5.7～12.6) | 0.43 | 0.38 | 3(-5.2～11.9) |
| 75-79 | 38 | 119 | 11.6*(2.3～21.7) | 0.32 | 0.85 | 10.1*(0.8～20.2) | 10 | 92 | 15.0*(8～22.4) | 0.08 | 0.66 | 13.6*(6.6～20.9) |
| 80-84 | 9 | 78 | - | 0.13 | 0.83 | - | 9 | 64 | - | 0.13 | 0.67 | - |
| 85+ | 8 | 25 | - | 0.20 | 0.39 | - | 0 | 33 | - | 0.00 | 0.52 | - |

Abbreviations: AAPC: average annual percentage change; CI: confidence interval.

*: statistically significance.

| **Table S3 Trends analysis of cases, incidence, deaths, and mortality of cervical cancer in China, by age, 2007-2016** | | | | | | | | | | | | |
| --- | --- | --- | --- | --- | --- | --- | --- | --- | --- | --- | --- | --- |
|  | **Cancer cases** | | | **Incidence** | | | **Cancer deaths** | | | **Mortality** | | |
| **Age group** | **2007** | **2016** | **AAPC (95% CI)** | **2007** | **2016** | **AAPC (95% CI)** | **2007** | **2016** | **AAPC (95% CI)** | **2007** | **2016** | **AAPC (95% CI)** |
| Total | 78510 | 113256 | 4.1*(3.3～4.9) | 8.50 | 11.39 | 3.4*(3.1～3.7) | 22457 | 35353 | 5.1*(3.1～7.0) | 2.37 | 3.40 | 4.9*(3.4～6.4) |
| 15-19 | 16 | 27 | 1.5(-11.3～16.1) | 0.03 | 0.08 | 6.6(-6.5～21.4) | 0 | 3 | - | 0.00 | 0.01 | - |
| 20-24 | 272 | 294 | -0.3(-6～5.7) | 0.61 | 0.65 | -0.3(-5.3～5) | 150 | 40 | -13.6*(-24.4～-1.3) | 0.34 | 0.09 | -15.1*(-26.6～-1.7) |
| 25-29 | 1095 | 1666 | 6.3*(3.2～9.4) | 2.49 | 2.64 | 1.4(-0.9～3.9) | 266 | 293 | 5.5(-2.7～14.5) | 0.60 | 0.46 | 0.9(-6.6～9) |
| 30-34 | 4359 | 4172 | -0.4(-2.6～1.8) | 8.87 | 7.96 | -1.3(-3.0～0.3) | 387 | 656 | 5.6*(2.6～8.7) | 0.79 | 1.25 | 4.5*(1.9～7.2) |
| 35-39 | 10478 | 6113 | -6.0*(-6.9～-5.1) | 16.18 | 12.85 | -2.3*(-3.4～-1.1) | 1514 | 906 | -4.7*(-7.1～-2.2) | 2.34 | 1.90 | -0.9(-3.7～1.9) |
| 40-44 | 17727 | 13422 | -2.5*(-4.4～-0.6) | 27.43 | 24.06 | -1.2(-2.5～0.2) | 2717 | 2552 | -0.6(-1.8～0.6) | 4.20 | 4.58 | 0.9(-0.3～2) |
| 45-49 | 9923 | 19597 | 6.5*(2.8～10.2) | 23.42 | 31.77 | 3.1*(0.8～5.4) | 1880 | 4660 | 9.0*(5.8～12.2) | 4.44 | 7.55 | 5.6*(3.5～7.7) |
| 50-54 | 11459 | 25392 | 9.4*(4.3～14.9) | 22.52 | 44.07 | 7.7*(6.6～8.7) | 3825 | 6338 | 8.1*(1.2～15.5) | 7.52 | 11.00 | 6.6*(3.1～10.2) |
| 55-59 | 8990 | 10863 | 2.3*(0.2～4.5) | 22.37 | 30.89 | 4.0*(2.8～5.3) | 2239 | 3527 | 2.7(-0.5～6) | 5.57 | 10.03 | 4.5*(1.1～8) |
| 60-64 | 5470 | 13060 | 11.0*(8.7～13.3) | 19.69 | 32.24 | 6.2*(4.8～7.7) | 1650 | 4877 | 10.6*(7～14.4) | 5.94 | 12.04 | 5.9*(2.9～9) |
| 65-69 | 3050 | 8012 | 12.7*(10.9～14.5) | 14.05 | 27.29 | 8.5*(7.1～9.9) | 2200 | 3565 | 9.5*(4.2～15.1) | 10.13 | 12.14 | 5.6*(1.2～10.2) |
| 70-74 | 2955 | 4777 | 5.8*(3.4～8.3) | 15.80 | 24.79 | 5.6*(4.1～7.1) | 2078 | 2692 | 4.6*(1.1～8.1) | 11.11 | 13.97 | 4.4*(1.7～7.2) |
| 75-79 | 1429 | 3140 | 9.3*(7.5～11.1) | 11.81 | 22.45 | 7.8*(5.9～9.8) | 1916 | 2412 | 5.3*(1.2～9.5) | 15.83 | 17.25 | 3.8(-0.5～8.3) |
| 80-84 | 856 | 1783 | 7.9*(6～9.7) | 12.17 | 18.92 | 4.3*(2.7～6) | 1110 | 1729 | 6.6*(3.1～10.3) | 15.77 | 18.36 | 3.2(-0.1～6.6) |
| 85+ | 352 | 927 | 2.5(-6.7～12.6) | 8.66 | 14.52 | -2.3(-10.7～6.9) | 525 | 1101 | 7.9*(5.1～10.8) | 12.90 | 17.25 | 2.7*(0.4～5.2) |

Abbreviations: AAPC: average annual percentage change; CI: confidence interval.

*: statistically significance.

| **Table S4 Trends analysis of cases, incidence, deaths, and mortality of uterine cancer in China, by age, 2007-2016** | | | | | | | | | | | | |
| --- | --- | --- | --- | --- | --- | --- | --- | --- | --- | --- | --- | --- |
|  | **Cancer cases** | | | **Incidence** | | | **Cancer deaths** | | | **Mortality** | | |
| **Age group** | **2007** | **2016** | **AAPC (95% CI)** | **2007** | **2016** | **AAPC (95% CI)** | **2007** | **2016** | **AAPC (95% CI)** | **2007** | **2016** | **AAPC (95% CI)** |
| Total | 50510 | 66228 | 3.3*(2.6～4.1) | 5.68 | 6.52 | 2.0*(1.1～2.9) | 18639 | 15891 | -1.7*(-3.1～-0.3) | 2.00 | 1.52 | -2.8*(-4.5～-1.1) |
| 15-19 | 49 | 6 | -11(-21～0.2) | 0.10 | 0.02 | -6.8(-17.2～5) | 0 | 4 | - | 0.00 | 0.01 | - |
| 20-24 | 47 | 102 | -1.1(-17.3～18.3) | 0.10 | 0.22 | 0.6(-12.7～15.9) | 38 | 38 | -0.2(-13.1～14.7) | 0.09 | 0.08 | 0(-11.2～12.7) |
| 25-29 | 462 | 591 | 2.6(-1.6～7.1) | 1.05 | 0.94 | -2(-6～2) | 112 | 82 | -8.7(-17.5～1) | 0.25 | 0.13 | -12.9*(-21.3～-3.7) |
| 30-34 | 887 | 957 | 3.2(-0.6～7.2) | 1.80 | 1.83 | 2.3(-1.4～6.1) | 261 | 117 | -8.8(-17.3～0.4) | 0.53 | 0.22 | -9.6*(-18.1～-0.1) |
| 35-39 | 2423 | 1862 | -2.6*(-4～-1.2) | 3.74 | 3.91 | 1.2(-0.3～2.8) | 563 | 235 | -10.2*(-15.7～-4.4) | 0.87 | 0.49 | -6.7*(-12.5～-0.4) |
| 40-44 | 4864 | 4837 | 0.8(-2.2～3.8) | 7.53 | 8.67 | 2.3(-0.5～5.1) | 1154 | 618 | -6.5*(-9.8～-3.1) | 1.79 | 1.11 | -5.2*(-8.2～-2.1) |
| 45-49 | 4875 | 10353 | 7.6*(3.5～11.7) | 11.51 | 16.78 | 4.1*(1.2～7.1) | 863 | 1399 | 2.3(-3.4～8.5) | 2.04 | 2.27 | -1(-5.6～3.8) |
| 50-54 | 11427 | 16544 | 4.7(-0.7～10.3) | 22.46 | 28.71 | 3.4*(1.8～5.1) | 2628 | 2511 | -1.1(-5.6～3.6) | 5.16 | 4.36 | -1.8(-3.6～0.1) |
| 55-59 | 10970 | 8353 | -1.8(-3.7～0.2) | 27.29 | 23.75 | -0.3(-1.7～1.1) | 3620 | 1467 | -6.7*(-10.3～-3) | 9.01 | 4.17 | -5.6*(-9.2～-1.9) |
| 60-64 | 5214 | 9997 | 7.7*(6.7～8.7) | 18.77 | 24.68 | 3.0*(1.5～4.6) | 1725 | 2720 | 4.2*(0.4～8.2) | 6.21 | 6.71 | -0.2(-4.1～3.9) |
| 65-69 | 3750 | 5985 | 6.6*(4.7～8.5) | 17.28 | 20.38 | 2.9*(1.8～3.9) | 2248 | 2232 | 1.1(-1.9～4.1) | 10.36 | 7.60 | -2.2*(-4.1～-0.3) |
| 70-74 | 3022 | 3145 | 0.1(-1.6～1.8) | 16.16 | 16.32 | 0.1(-0.9～1.1) | 2081 | 1589 | -2.9*(-5.6～-0.1) | 11.13 | 8.25 | -2.8*(-5.1～-0.4) |
| 75-79 | 1389 | 1950 | 1.4(-1.9～4.9) | 11.48 | 13.95 | 0.2(-2.8～3.2) | 1659 | 1212 | -4.3*(-6.3～-2.2) | 13.71 | 8.67 | -5.5*(-7.2～-3.8) |
| 80-84 | 578 | 1077 | 3.6(-0.4～7.7) | 8.21 | 11.43 | 0.2(-3.6～4.2) | 1072 | 974 | -1(-4～2.1) | 15.23 | 10.34 | -4.2*(-7～-1.2) |
| 85+ | 517 | 460 | -1.2(-3～0.6) | 12.70 | 7.21 | -5.8*(-7.6～-4.1) | 616 | 694 | -0.3(-3.8～3.2) | 15.14 | 10.88 | -5.0*(-8.5～-1.5) |

Abbreviations: AAPC: average annual percentage change; CI: confidence interval.

*: statistically significance.

| **Table S5 Trends analysis of cases, incidence, deaths, and mortality of ovarian cancer in China, by age, 2007-2016** | | | | | | | | | | | | |
| --- | --- | --- | --- | --- | --- | --- | --- | --- | --- | --- | --- | --- |
|  | **Cancer cases** | | | **Incidence** | | | **Cancer deaths** | | | **Mortality** | | |
| **Age group** | **2007** | **2016** | **AAPC (95% CI)** | **2007** | **2016** | **AAPC (95% CI)** | **2007** | **2016** | **AAPC (95% CI)** | **2007** | **2016** | **AAPC (95% CI)** |
| Total | 43023 | 53490 | 2.4*(1.4～3.5) | 5.11 | 5.41 | 0.9*(0.2～1.7) | 16355 | 24953 | 4.5*(3.2～5.8) | 1.87 | 3.40 | 2.4*(1.9～2.9) |
| 15-19 | 297 | 394 | 1.9(-3.1～7.2) | 0.58 | 1.16 | 6.9*(1.9～12.2) | 16 | 30 | 1.0(-13.5～17.9) | 0.03 | 0.09 | 6.1(-8.7～23.2) |
| 20-24 | 823 | 813 | 0(-7.4～7.9) | 1.84 | 1.79 | 0(-4.1～4.2) | 12 | 71 | -7.9(-18.6～4.1) | 0.03 | 0.16 | -7.9(-17.5～2.8) |
| 25-29 | 1059 | 1747 | 7.5*(4.2～10.9) | 2.41 | 2.76 | 2.6*(0～5.4) | 141 | 243 | 4.3(-2.4～11.5) | 0.32 | 0.38 | -0.3(-6.5～6.3) |
| 30-34 | 1183 | 1517 | 1.9(-0.9～4.8) | 2.41 | 2.90 | 0.9(-2.2～4.1) | 79 | 247 | 5.1(-2.8～13.6) | 0.16 | 0.47 | 3.8(-4.1～12.5) |
| 35-39 | 2888 | 1702 | -4.8*(-6.5～-3.0) | 4.46 | 3.58 | -1.0(-3.0～1.0) | 574 | 454 | -3.7(-8.4～1.3) | 0.89 | 0.96 | 0.1(-4.5～4.9) |
| 40-44 | 4062 | 3967 | -0.3(-2.2～1.7) | 6.29 | 7.11 | 1.2(-0.2～2.7) | 1030 | 1212 | 1.8(-0.6～4.2) | 1.59 | 2.17 | 3.3*(1.0～5.7) |
| 45-49 | 4634 | 7584 | 4.4*(2.0～6.9) | 10.94 | 12.29 | 1.0(-0.4～2.4) | 1424 | 2415 | 5.7*(3.0～8.5) | 3.36 | 3.91 | 2.2*(0.3～4.2) |
| 50-54 | 8330 | 9962 | 1.4(-4.0～7.1) | 16.37 | 17.29 | 0.5(-1.3～2.3) | 2664 | 4082 | 3.9(-1.6～9.6) | 5.24 | 7.08 | 2.7*(0.9～4.5) |
| 55-59 | 6002 | 5305 | -1.1(-3.6～1.5) | 14.93 | 15.08 | 0.4(-1.0～1.9) | 2513 | 2569 | 1.2(-2.0～4.5) | 6.25 | 7.30 | 2.8*(0.1～5.5) |
| 60-64 | 4648 | 7501 | 6.7*(4.9～8.5) | 16.73 | 18.52 | 2.2*(1.0～3.4) | 1903 | 3981 | 8.0*(6.6～9.5) | 6.85 | 9.83 | 3.4*(2.1～4.7) |
| 65-69 | 2820 | 5261 | 7.5*(5.9～9.1) | 12.99 | 17.92 | 3.7*(2.7～4.7) | 1457 | 3313 | 9.0*(6.7～11.3) | 6.71 | 11.29 | 5.0*(3.5～6.6) |
| 70-74 | 3043 | 3349 | 1.9(-0.7～4.6) | 16.28 | 17.38 | 1.9*(0～3.8) | 2256 | 2338 | 1.2(-2.2～4.8) | 12.06 | 12.13 | 1.2(-1.2～3.6) |
| 75-79 | 1732 | 2162 | 2.1*(0.8～3.5) | 14.31 | 15.46 | 0.8(-0.5～2.1) | 1275 | 1883 | 3.5*(1.9～5.2) | 10.53 | 13.47 | 2.2*(0.9～3.5) |
| 80-84 | 825 | 1365 | 5.8*(3.3～8.4) | 11.72 | 14.49 | 2.4*(0.1～4.7) | 683 | 1427 | 7.3*(4.3～10.4) | 9.70 | 15.15 | 3.8*(1.0～6.7) |
| 85+ | 337 | 558 | 3.7*(0～7.6) | 8.29 | 8.75 | -1.2(-4.3～2.1) | 329 | 667 | 7.7*(2.7～12.8) | 8.08 | 10.45 | 2.4(-2.5～7.6) |

Abbreviations: AAPC: average annual percentage change; CI: confidence interval.

*: statistically significance.

| **Table S6 Trends analysis of cases, incidence, deaths, and mortality of other gynecological cancer in China, by age, 2007-2016** | | | | | | | | | | | | |
| --- | --- | --- | --- | --- | --- | --- | --- | --- | --- | --- | --- | --- |
|  | **Cancer cases** | | | **Incidence** | | | **Cancer deaths** | | | **Mortality** | | |
| **Age group** | **2007** | **2016** | **AAPC (95% CI)** | **2007** | **2016** | **AAPC (95% CI)** | **2007** | **2016** | **AAPC (95% CI)** | **2007** | **2016** | **AAPC (95% CI)** |
| Total | 2585 | 4437 | 3.6*(1.4～5.9) | 0.32 | 0.46 | 1.8(-0.6～4.3) | 418 | 1357 | 9.7*(4.8～14.8) | 0.05 | 0.13 | 7.3*(2.3～12.5) |
| 15-19 | 16 | 42 | -8.4(-21.7～7.2) | 0.03 | 0.12 | -3.9(-17.7～12.1) | 0 | 4 | - | 0.00 | 0.01 | - |
| 20-24 | 58 | 86 | -4.9(-19.2～11.9) | 0.13 | 0.19 | -4.6(-15.8～8.0) | 0 | 6 | - | 0.00 | 0.01 | - |
| 25-29 | 174 | 272 | 3.7(-2.6～10.3) | 0.40 | 0.43 | -0.9(-6.6～5.1) | 0 | 40 | - | 0.00 | 0.06 | - |
| 30-34 | 211 | 192 | -3.3(-10.1～3.9) | 0.43 | 0.37 | -4.3(-10.9～2.9) | 0 | 14 | - | 0.00 | 0.03 | - |
| 35-39 | 192 | 160 | 0.8(-5.2～7.2) | 0.30 | 0.34 | 4.7(-1.6～11.4) | 0 | 21 | - | 0.00 | 0.04 | - |
| 40-44 | 146 | 358 | 1.2(-5.3～8.2) | 0.23 | 0.64 | 2.8(-4.0～10.0) | 30 | 59 | 4.3(-6.0～15.9) | 0.05 | 0.10 | 6.0(-4.4～17.5) |
| 45-49 | 236 | 540 | 3.6(-4.9～12.9) | 0.56 | 0.88 | 0.4(-7.1～8.6) | 32 | 113 | 10.2*(1.7～19.4) | 0.08 | 0.18 | 6.9(-0.5～14.9) |
| 50-54 | 438 | 802 | 1.8(-5.1～9.3) | 0.86 | 1.39 | 0.7(-3.8～5.5) | 12 | 222 | 13.2*(4.2～23.0) | 0.02 | 0.38 | 10.1*(1.2～19.7) |
| 55-59 | 356 | 394 | 0(-5.1～5.4) | 0.89 | 1.12 | 1.5(-2.8～6.1) | 41 | 108 | 5.6(-7.5～20.5) | 0.10 | 0.31 | 7.9(-4.5～22.0) |
| 60-64 | 281 | 585 | 10.1*(6.9～13.4) | 1.01 | 1.45 | 5.4*(2.7～8.1) | 63 | 203 | 11.1*(4.0～18.7) | 0.23 | 0.50 | 6.4*(0.1～13.2) |
| 65-69 | 140 | 486 | 14.7*(11.2～18.4) | 0.64 | 1.66 | 10.4*(7.6～13.2) | 35 | 214 | 20.8*(13.2～28.9) | 0.16 | 0.73 | 16.0*(8.5～24.1) |
| 70-74 | 215 | 204 | 3.4(-2.5～9.7) | 1.15 | 1.06 | 3.4(-2.3～9.5) | 91 | 142 | 7.0*(1.2～13.2) | 0.49 | 0.74 | 6.8*(0.9～13.0) |
| 75-79 | 94 | 147 | 0.8(-5.3～7.3) | 0.78 | 1.05 | -0.4(-6.2～5.7) | 38 | 119 | 6.2*(0～12.8) | 0.32 | 0.85 | 5.0(-0.9～11.1) |
| 80-84 | 18 | 114 | 5.8(-5.0～17.8) | 0.26 | 1.21 | 2.3(-8.1～13.9) | 18 | 64 | 4.5(-4.0～13.8) | 0.26 | 0.68 | 1.1(-7.2～10.2) |
| 85+ | 8 | 42 | 9.3(-3.9～24.3) | 0.20 | 0.66 | 4.1(-8.4～18.4) | 57 | 24 | 2.5(-9.6～16.1) | 1.41 | 0.37 | -2.4(-13.9～10.7) |

Abbreviations: AAPC: average annual percentage change; CI: confidence interval.

*: statistically significance.

**Table S7 Projection of gynecological cancer cases and incidence in China, by geographical area, 2017-2030**

|  | **Cancer cases** | | | | | | |  | **ASIR** | | | | | | |
| --- | --- | --- | --- | --- | --- | --- | --- | --- | --- | --- | --- | --- | --- | --- | --- |
| **Year** | **Vulva** | **Vagina** | **Cervix** | **Uterus** | **Ovary** | **Other GC** | **Total** |  | **Vulva** | **Vagina** | **Cervix** | **Uterus** | **Ovary** | **Other GC** | **Total** |
| **Total** |  |  |  |  |  |  |  |  |  |  |  |  |  |  |  |
| 2017 | 2944 | 1654 | 115603 | 68830 | 53336 | 4214 | 246581 |  | 0.28 | 0.16 | 11.79 | 6.87 | 5.46 | 0.43 | 24.99 |
| 2018 | 3071 | 1743 | 120756 | 71313 | 54830 | 4327 | 256040 |  | 0.29 | 0.17 | 12.20 | 7.02 | 5.52 | 0.44 | 25.64 |
| 2019 | 3205 | 1836 | 126173 | 73888 | 56365 | 4444 | 265911 |  | 0.29 | 0.17 | 12.62 | 7.16 | 5.59 | 0.44 | 26.27 |
| 2020 | 3346 | 1935 | 131869 | 76557 | 57945 | 4565 | 276217 |  | 0.30 | 0.18 | 13.06 | 7.31 | 5.66 | 0.44 | 26.95 |
| 2021 | 3494 | 2040 | 137859 | 79325 | 59570 | 4689 | 286977 |  | 0.30 | 0.18 | 13.52 | 7.47 | 5.73 | 0.45 | 27.65 |
| 2022 | 3650 | 2150 | 144160 | 82195 | 61240 | 4818 | 298213 |  | 0.31 | 0.19 | 13.99 | 7.62 | 5.80 | 0.45 | 28.36 |
| 2023 | 3813 | 2265 | 150788 | 85169 | 62958 | 4951 | 309944 |  | 0.32 | 0.19 | 14.47 | 7.78 | 5.87 | 0.45 | 29.08 |
| 2024 | 3986 | 2387 | 157763 | 88255 | 64725 | 5089 | 322205 |  | 0.33 | 0.20 | 14.97 | 7.95 | 5.95 | 0.46 | 29.86 |
| 2025 | 4167 | 2516 | 165103 | 91454 | 66542 | 5230 | 335012 |  | 0.33 | 0.21 | 15.49 | 8.11 | 6.02 | 0.46 | 30.62 |
| 2026 | 4358 | 2651 | 172830 | 94772 | 68411 | 5377 | 348399 |  | 0.34 | 0.21 | 16.03 | 8.28 | 6.09 | 0.46 | 31.41 |
| 2027 | 4560 | 2795 | 180964 | 98211 | 70333 | 5529 | 362392 |  | 0.35 | 0.22 | 16.59 | 8.46 | 6.17 | 0.47 | 32.26 |
| 2028 | 4772 | 2945 | 189530 | 101779 | 72310 | 5686 | 377022 |  | 0.35 | 0.23 | 17.16 | 8.63 | 6.25 | 0.47 | 33.09 |
| 2029 | 4996 | 3104 | 198551 | 105479 | 74342 | 5848 | 392320 |  | 0.36 | 0.23 | 17.76 | 8.82 | 6.32 | 0.47 | 33.96 |
| 2030 | 5231 | 3271 | 208054 | 109314 | 76433 | 6015 | 408318 |  | 0.37 | 0.24 | 18.38 | 9.00 | 6.40 | 0.48 | 34.87 |
| **Urban** |  |  |  |  |  |  |  |  |  |  |  |  |  |  |  |
| 2017 | 1676 | 960 | 64511 | 38921 | 31738 | 2607 | 140413 |  | 0.29 | 0.17 | 11.24 | 6.83 | 5.69 | 0.46 | 24.68 |
| 2018 | 1722 | 1012 | 68373 | 40497 | 32543 | 2703 | 146850 |  | 0.29 | 0.18 | 11.55 | 6.86 | 5.63 | 0.46 | 24.97 |
| 2019 | 1769 | 1066 | 72467 | 42138 | 33368 | 2803 | 153611 |  | 0.29 | 0.18 | 11.86 | 6.88 | 5.57 | 0.46 | 25.24 |
| 2020 | 1818 | 1124 | 76806 | 43844 | 34214 | 2906 | 160712 |  | 0.28 | 0.18 | 12.19 | 6.90 | 5.50 | 0.46 | 25.51 |
| 2021 | 1867 | 1185 | 81405 | 45620 | 35082 | 3013 | 168172 |  | 0.28 | 0.19 | 12.52 | 6.93 | 5.44 | 0.46 | 25.82 |
| 2022 | 1919 | 1249 | 86279 | 47468 | 35972 | 3124 | 176011 |  | 0.28 | 0.19 | 12.87 | 6.95 | 5.38 | 0.46 | 26.13 |
| 2023 | 1971 | 1316 | 91445 | 49390 | 36884 | 3239 | 184245 |  | 0.28 | 0.19 | 13.22 | 6.97 | 5.32 | 0.46 | 26.44 |
| 2024 | 2025 | 1387 | 96920 | 51391 | 37819 | 3359 | 192901 |  | 0.27 | 0.20 | 13.58 | 7.00 | 5.27 | 0.45 | 26.77 |
| 2025 | 2080 | 1462 | 102723 | 53472 | 38778 | 3482 | 201997 |  | 0.27 | 0.20 | 13.95 | 7.02 | 5.21 | 0.45 | 27.10 |
| 2026 | 2137 | 1541 | 108874 | 55638 | 39762 | 3611 | 211563 |  | 0.27 | 0.21 | 14.34 | 7.05 | 5.15 | 0.45 | 27.47 |
| 2027 | 2196 | 1625 | 115392 | 57891 | 40770 | 3744 | 221618 |  | 0.27 | 0.21 | 14.73 | 7.07 | 5.09 | 0.45 | 27.82 |
| 2028 | 2256 | 1712 | 122301 | 60236 | 41804 | 3882 | 232191 |  | 0.26 | 0.21 | 15.13 | 7.09 | 5.04 | 0.45 | 28.18 |
| 2029 | 2318 | 1805 | 129624 | 62676 | 42864 | 4025 | 243312 |  | 0.26 | 0.22 | 15.55 | 7.12 | 4.98 | 0.45 | 28.58 |
| 2030 | 2381 | 1902 | 137385 | 65214 | 43951 | 4173 | 255006 |  | 0.26 | 0.22 | 15.97 | 7.14 | 4.93 | 0.45 | 28.97 |
| **Rural** |  |  |  |  |  |  |  |  |  |  |  |  |  |  |  |
| 2017 | 1268 | 694 | 51092 | 29909 | 21598 | 1607 | 106168 |  | 0.28 | 0.15 | 12.36 | 6.92 | 5.25 | 0.40 | 25.36 |
| 2018 | 1349 | 731 | 52383 | 30816 | 22287 | 1624 | 109190 |  | 0.30 | 0.16 | 12.88 | 7.21 | 5.48 | 0.41 | 26.44 |
| 2019 | 1436 | 770 | 53706 | 31750 | 22997 | 1641 | 112300 |  | 0.32 | 0.16 | 13.43 | 7.50 | 5.72 | 0.42 | 27.55 |
| 2020 | 1528 | 811 | 55063 | 32713 | 23731 | 1659 | 115505 |  | 0.34 | 0.17 | 13.99 | 7.81 | 5.97 | 0.43 | 28.71 |
| 2021 | 1627 | 855 | 56454 | 33705 | 24488 | 1676 | 118805 |  | 0.37 | 0.18 | 14.58 | 8.13 | 6.23 | 0.44 | 29.93 |
| 2022 | 1731 | 901 | 57881 | 34727 | 25268 | 1694 | 122202 |  | 0.39 | 0.19 | 15.2 | 8.46 | 6.50 | 0.44 | 31.18 |
| 2023 | 1842 | 949 | 59343 | 35779 | 26074 | 1712 | 125699 |  | 0.42 | 0.20 | 15.84 | 8.81 | 6.79 | 0.45 | 32.51 |
| 2024 | 1961 | 1000 | 60843 | 36864 | 26906 | 1730 | 129304 |  | 0.45 | 0.21 | 16.51 | 9.17 | 7.09 | 0.46 | 33.89 |
| 2025 | 2087 | 1054 | 62380 | 37982 | 27764 | 1748 | 133015 |  | 0.48 | 0.22 | 17.2 | 9.55 | 7.40 | 0.47 | 35.32 |
| 2026 | 2221 | 1110 | 63956 | 39134 | 28649 | 1766 | 136836 |  | 0.52 | 0.23 | 17.93 | 9.94 | 7.73 | 0.48 | 36.83 |
| 2027 | 2364 | 1170 | 65572 | 40320 | 29563 | 1785 | 140774 |  | 0.55 | 0.24 | 18.68 | 10.35 | 8.06 | 0.49 | 38.37 |
| 2028 | 2516 | 1233 | 67229 | 41543 | 30506 | 1804 | 144831 |  | 0.59 | 0.25 | 19.47 | 10.77 | 8.42 | 0.50 | 40.00 |
| 2029 | 2678 | 1299 | 68927 | 42803 | 31478 | 1823 | 149008 |  | 0.64 | 0.26 | 20.29 | 11.21 | 8.79 | 0.51 | 41.70 |
| 2030 | 2850 | 1369 | 70669 | 44100 | 32482 | 1842 | 153312 |  | 0.68 | 0.27 | 21.15 | 11.67 | 9.18 | 0.52 | 43.47 |

Abbreviations: Abbreviations: ASIR, age-standardized incidence rate; ASMR, age-standardized mortality rate; GC: Gynecological cancer.

**Table S8 Projection of gynecological cancer deaths and mortality in China, by geographical area, 2017-2030**

|  | **Cancer deaths** | | | | | | |  | **ASMR** | | | | | | |
| --- | --- | --- | --- | --- | --- | --- | --- | --- | --- | --- | --- | --- | --- | --- | --- |
| **Year** | **Vulva** | **Vagina** | **Cervix** | **Uterus** | **Ovary** | **Other GC** | **Total** |  | **Vulva** | **Vagina** | **Cervix** | **Uterus** | **Ovary** | **Other GC** | **Total** |
| **Total** |  |  |  |  |  |  |  |  |  |  |  |  |  |  |  |
| 2017 | 1148 | 680 | 36770 | 15642 | 24917 | 1504 | 80661 |  | 0.10 | 0.06 | 3.52 | 1.52 | 2.36 | 0.14 | 7.70 |
| 2018 | 1241 | 736 | 39823 | 15467 | 26135 | 1630 | 85032 |  | 0.11 | 0.07 | 3.73 | 1.48 | 2.42 | 0.15 | 7.96 |
| 2019 | 1343 | 796 | 43200 | 15303 | 27411 | 1767 | 89820 |  | 0.11 | 0.07 | 3.95 | 1.44 | 2.48 | 0.16 | 8.21 |
| 2020 | 1453 | 861 | 46935 | 15148 | 28750 | 1917 | 95064 |  | 0.12 | 0.07 | 4.19 | 1.40 | 2.54 | 0.17 | 8.49 |
| 2021 | 1572 | 933 | 51072 | 15002 | 30154 | 2081 | 100814 |  | 0.12 | 0.08 | 4.43 | 1.36 | 2.60 | 0.18 | 8.77 |
| 2022 | 1702 | 1010 | 55656 | 14868 | 31627 | 2260 | 107123 |  | 0.13 | 0.08 | 4.70 | 1.32 | 2.67 | 0.19 | 9.09 |
| 2023 | 1844 | 1094 | 60742 | 14741 | 33172 | 2456 | 114049 |  | 0.14 | 0.08 | 4.97 | 1.28 | 2.78 | 0.20 | 9.45 |
| 2024 | 1998 | 1184 | 66387 | 14624 | 34792 | 2672 | 121657 |  | 0.15 | 0.09 | 5.27 | 1.25 | 2.80 | 0.21 | 9.77 |
| 2025 | 2165 | 1283 | 72657 | 14515 | 36491 | 2907 | 130018 |  | 0.15 | 0.09 | 5.58 | 1.22 | 2.87 | 0.22 | 10.13 |
| 2026 | 2346 | 1389 | 79627 | 14414 | 38274 | 3164 | 139214 |  | 0.16 | 0.10 | 5.91 | 1.18 | 2.94 | 0.23 | 10.52 |
| 2027 | 2544 | 1505 | 87380 | 14322 | 40144 | 3447 | 149342 |  | 0.17 | 0.10 | 6.26 | 1.15 | 3.01 | 0.25 | 10.94 |
| 2028 | 2759 | 1632 | 96007 | 14238 | 42104 | 3757 | 160497 |  | 0.18 | 0.11 | 6.63 | 1.12 | 3.09 | 0.26 | 11.39 |
| 2029 | 2992 | 1768 | 105613 | 14162 | 44161 | 4097 | 172793 |  | 0.19 | 0.11 | 7.02 | 1.09 | 3.17 | 0.27 | 11.85 |
| 2030 | 3247 | 1916 | 116315 | 14093 | 46318 | 4471 | 186360 |  | 0.20 | 0.12 | 7.44 | 1.06 | 3.24 | 0.29 | 12.35 |
| **Urban** |  |  |  |  |  |  |  |  |  |  |  |  |  |  |  |
| 2017 | 649 | 418 | 20417 | 8060 | 15359 | 835 | 45738 |  | 0.11 | 0.07 | 3.46 | 1.42 | 2.65 | 0.14 | 7.85 |
| 2018 | 691 | 457 | 22899 | 8151 | 16110 | 886 | 49194 |  | 0.11 | 0.07 | 3.73 | 1.38 | 2.67 | 0.15 | 8.11 |
| 2019 | 737 | 499 | 25684 | 8244 | 16897 | 939 | 53000 |  | 0.11 | 0.07 | 4.02 | 1.35 | 2.69 | 0.15 | 8.39 |
| 2020 | 785 | 545 | 28807 | 8337 | 17723 | 996 | 57193 |  | 0.11 | 0.08 | 4.34 | 1.32 | 2.71 | 0.15 | 8.71 |
| 2021 | 837 | 596 | 32310 | 8431 | 18589 | 1056 | 61819 |  | 0.12 | 0.08 | 4.67 | 1.29 | 2.73 | 0.15 | 9.04 |
| 2022 | 892 | 651 | 36238 | 8527 | 19497 | 1119 | 66924 |  | 0.12 | 0.08 | 5.04 | 1.27 | 2.76 | 0.15 | 9.42 |
| 2023 | 951 | 712 | 40645 | 8623 | 20450 | 1187 | 72568 |  | 0.12 | 0.09 | 5.43 | 1.24 | 2.78 | 0.15 | 9.81 |
| 2024 | 1014 | 778 | 45587 | 8721 | 21449 | 1259 | 78808 |  | 0.12 | 0.09 | 5.86 | 1.21 | 2.80 | 0.16 | 10.24 |
| 2025 | 1081 | 850 | 51130 | 8820 | 22497 | 1335 | 85713 |  | 0.13 | 0.10 | 6.32 | 1.18 | 2.82 | 0.16 | 10.71 |
| 2026 | 1152 | 929 | 57347 | 8919 | 23597 | 1415 | 93359 |  | 0.13 | 0.10 | 6.81 | 1.16 | 2.84 | 0.16 | 11.20 |
| 2027 | 1228 | 1015 | 64321 | 9020 | 24750 | 1500 | 101834 |  | 0.13 | 0.10 | 7.34 | 1.13 | 2.87 | 0.16 | 11.73 |
| 2028 | 1309 | 1110 | 72142 | 9122 | 25959 | 1591 | 111233 |  | 0.13 | 0.11 | 7.92 | 1.11 | 2.89 | 0.16 | 12.32 |
| 2029 | 1395 | 1213 | 80914 | 9226 | 27228 | 1687 | 121663 |  | 0.14 | 0.11 | 8.54 | 1.08 | 2.91 | 0.16 | 12.94 |
| 2030 | 1487 | 1325 | 90752 | 9330 | 28558 | 1789 | 133241 |  | 0.14 | 0.12 | 9.21 | 1.06 | 2.93 | 0.17 | 13.63 |
| **Rural** |  |  |  |  |  |  |  |  |  |  |  |  |  |  |  |
| 2017 | 499 | 262 | 16353 | 7582 | 9558 | 669 | 34923 |  | 0.10 | 0.05 | 3.59 | 1.62 | 2.08 | 0.14 | 7.58 |
| 2018 | 550 | 279 | 16924 | 7316 | 10025 | 744 | 35838 |  | 0.11 | 0.06 | 3.75 | 1.57 | 2.18 | 0.16 | 7.83 |
| 2019 | 606 | 297 | 17516 | 7059 | 10514 | 828 | 36820 |  | 0.12 | 0.06 | 3.91 | 1.52 | 2.29 | 0.18 | 8.08 |
| 2020 | 668 | 316 | 18128 | 6811 | 11027 | 921 | 37871 |  | 0.13 | 0.07 | 4.08 | 1.47 | 2.41 | 0.20 | 8.36 |
| 2021 | 735 | 337 | 18762 | 6571 | 11565 | 1025 | 38995 |  | 0.14 | 0.07 | 4.26 | 1.43 | 2.53 | 0.22 | 8.65 |
| 2022 | 810 | 359 | 19418 | 6341 | 12130 | 1141 | 40199 |  | 0.15 | 0.07 | 4.44 | 1.38 | 2.66 | 0.25 | 8.95 |
| 2023 | 893 | 382 | 20097 | 6118 | 12722 | 1269 | 41481 |  | 0.17 | 0.08 | 4.63 | 1.34 | 2.79 | 0.28 | 9.29 |
| 2024 | 984 | 406 | 20800 | 5903 | 13343 | 1413 | 42849 |  | 0.19 | 0.08 | 4.83 | 1.29 | 2.93 | 0.31 | 9.63 |
| 2025 | 1084 | 433 | 21527 | 5695 | 13994 | 1572 | 44305 |  | 0.21 | 0.09 | 5.04 | 1.25 | 3.08 | 0.35 | 10.02 |
| 2026 | 1194 | 460 | 22280 | 5495 | 14677 | 1749 | 45855 |  | 0.23 | 0.09 | 5.26 | 1.21 | 3.23 | 0.39 | 10.41 |
| 2027 | 1316 | 490 | 23059 | 5302 | 15394 | 1947 | 47508 |  | 0.25 | 0.10 | 5.48 | 1.18 | 3.39 | 0.43 | 10.83 |
| 2028 | 1450 | 522 | 23865 | 5116 | 16145 | 2166 | 49264 |  | 0.27 | 0.11 | 5.72 | 1.14 | 3.56 | 0.48 | 11.28 |
| 2029 | 1597 | 555 | 24699 | 4936 | 16933 | 2410 | 51130 |  | 0.30 | 0.11 | 5.97 | 1.10 | 3.74 | 0.54 | 11.76 |
| 2030 | 1760 | 591 | 25563 | 4763 | 17760 | 2682 | 53119 |  | 0.33 | 0.12 | 6.22 | 1.07 | 3.93 | 0.60 | 12.27 |

Abbreviations: Abbreviations: ASMR, age-standardized mortality rate; GC, gynecological cancer.
